# Spatial and temporal genetic dynamics of the grasshopper *Oedaleus decorus* revealed by museum genomics

**DOI:** 10.1002/ece3.3699

**Published:** 2017-12-29

**Authors:** Sarah Schmid, Samuel Neuenschwander, Camille Pitteloud, Gerald Heckel, Mila Pajkovic, Raphaël Arlettaz, Nadir Alvarez

**Affiliations:** ^1^ Department of Ecology and Evolution University of Lausanne Lausanne Switzerland; ^2^ Vital‐IT Swiss Institute of Bioinformatics University of Lausanne Lausanne Switzerland; ^3^ Department of Environmental Systems Science Eidgenössische Technische Hochschule Zürich Zürich Switzerland; ^4^ Institute of Ecology and Evolution University of Bern Bern Switzerland; ^5^ Natural History Museum of Geneva Geneva Switzerland

**Keywords:** conservation genetics, hybridization capture RAD, phylogeography, population genetics

## Abstract

Analyzing genetic variation through time and space is important to identify key evolutionary and ecological processes in populations. However, using contemporary genetic data to infer the dynamics of genetic diversity may be at risk of a bias, as inferences are performed from a set of extant populations, setting aside unavailable, rare, or now extinct lineages. Here, we took advantage of new developments in next‐generation sequencing to analyze the spatial and temporal genetic dynamics of the grasshopper *Oedaleus decorus*, a steppic Southwestern‐Palearctic species. We applied a recently developed hybridization capture (hyRAD) protocol that allows retrieving orthologous sequences even from degraded DNA characteristic of museum specimens. We identified single nucleotide polymorphisms in 68 historical and 51 modern samples in order to (i) unravel the spatial genetic structure across part of the species distribution and (ii) assess the loss of genetic diversity over the past century in Swiss populations. Our results revealed (i) the presence of three potential glacial refugia spread across the European continent and converging spatially in the Alpine area. In addition, and despite a limited population sample size, our results indicate (ii) a loss of allelic richness in contemporary Swiss populations compared to historical populations, whereas levels of expected heterozygosities were not significantly different. This observation is compatible with an increase in the bottleneck magnitude experienced by central European populations of *O. decorus* following human‐mediated land‐use change impacting steppic habitats. Our results confirm that application of hyRAD to museum samples produces valuable information to study genetic processes across time and space.

## 1 INTRODUCTION

Understanding processes shaping the genetic variation in species through space and time is crucial in various fields of biology, ranging from informing conservation decisions (e.g., Inoue, Lang, & Berg, 2015; Mukesh, Sharma, Shukla, & Sathyakumar, 2015) and unraveling the demographic history of species (e.g., Gong et al., 2015; Tsuda, Nakao, Ide, & Tsumura, 2015; Vera Escalona, Habit, & Ruzzante, 2015) to disentangling key evolutionary processes (e.g., Capblancq, Després, Rioux, & Mavárez, 2015). Investigating genetic variation through space has proven valuable to unravel the role of environment in genetic divergence (Björklund, Alonso, & Edelaar, 2013; D'Amen, Zimmermann, & Pearman, 2013). Such information is of high interest because a species’ genetic structure (i) informs about a species’ response to climatic oscillations as revealed by large‐scale spatial clustering and inference of potential glacial refugia in which differentiated lineages persisted (Taberlet, Fumagalli, Wust‐Saucy, & Cosson, 1998), and bears the signature of (ii) its recent demographic history, as well as of (iii) processes involved in local adaptation. Because evolution is a change over time, documenting temporal changes in genetic variation is central to numerous studies (Park et al., 2015; Ramakrishnan & Hadly, 2009; Spurgin et al., 2014), as those aiming to identify species’ responses to climate change (Serra‐Varela et al., 2015; Soltis, Morris, McLachlan, Manos, & Soltis, 2006) or the ecological factors linked with population divergence and speciation (Richards, Carstens, & Knowles, 2007a). In conservation biology, for instance, the level of genetic diversity in threatened species is of concern because genetic variation is needed for ongoing and future adaptive evolution and to cope with environmental changes (Frankham et al., 2002).

In contrast, investigating genetic structuring through time has received less attention, potentially because of the difficulty to collect population‐level samples through time series. Indeed, in most cases, the effects of past processes are inferred from genetic information obtained from modern populations, either by describing within and among population genetic variation, or using more sophisticated model‐based coalescence approaches (Shafer et al., 2015). Contemporary genetic data have revealed powerful to detect population decline (e.g., Rodríguez‐Zárate, Rocha‐Olivares, & Beheregaray, 2013; Salmona et al., 2012) and to elucidate population history of young species (e.g., Li & Durbin, 2011). However, genetic structure inferred using contemporary samples mainly reflects recent demographic and evolutionary processes (Hansen et al., 2009). Moreover, different processes can produce the same signature in modern data. Indeed, recent or older divergence followed by recent gene flow both result in weakly differentiated populations (Ramakrishnan & Hadly, 2009). Additionally, events such as genetic bottlenecks may eliminate past genetic signatures, rendering ancient demographic processes difficult to detect (Heled & Drummond, 2008). Overcoming these difficulties might be achieved by analyzing historical genetic data in order to get a direct insight into the demographic and evolutionary history of populations (Schwartz et al., 2007). This might allow, for instance, understanding the causes of temporal fluctuations in effective population size as indirectly identified using genetic diversity estimates and may provide information about the response of populations to past events such as human‐driven disturbances (e.g., Hartmann, Schaefer, & Segelbacher, 2014; Poudel, Moeller, Li, Shah, & Gao, 2014).

Museum specimens represent an underused resource to reconstruct the evolutionary history of species in space and time (Habel, Husemann, Finger, Danley, & Zachos, 2014; Wandeler, Hoeck, & Keller, 2007). With an estimated 2.5–3 billion biological specimens collected and accumulated worldwide over about 300 years, museum samples have the potential to significantly increase the amount of available spatial and temporal data (Krishtalka & Humphrey, 2000; O'Connell, Gilbert, & Hatfield, 2004; Pyke & Ehrlich, 2010). Genetic analyses of museum specimens may allow exploring unique biological collections, comprising both extinct and rare lineages, which are impossible or difficult to collect. They also enable the incorporation of samples from remote or currently inaccessible locations (Pyke & Ehrlich, 2010). Not only does DNA extracted from museum specimens allow reconstructing more accurate species’ spatial genetic structures (e.g., Perktaş & Quintero, 2013) but it provides direct information on past genetic diversity (Ramakrishnan, Hadly, & Mountain, 2005). Various studies successfully took advantage of museum specimens to unravel pathogen origin and dynamics (see Tsangaras & Greenwood, 2012 for a review) or to study population genetics (see Wandeler et al., 2007 for a review).

To date, however, most genetic analyses based on museum samples rely on PCR amplification of targeted genes (e.g., Habel et al., 2009; La Haye, Neumann, & Koelewijn, 2012; Pacioni et al., 2015). Such analyses usually incorporate a limited number of samples because of the degraded nature of their DNA. Samples with highly degraded DNA content might hold valuable genetic information, but DNA fragments shorter than the region targeted by the PCR primers cannot be amplified (Tin, Economo, & Mikheyev, 2014). The recent advent of next‐generation sequencing combined with target‐enrichment methods provides the opportunity to overcome this difficulty and to genotype genome‐wide single nucleotide polymorphism (SNP) in historical samples (Hofreiter et al., 2015). However, most methods developed so far are difficult to use in nonmodel organisms.

Here, we combined a classical shotgun protocol with hybridization capture of restriction site‐associated DNA loci (hyRAD)—a recently developed technique using fragments generated by RAD as baits to capture homologous sequences generated by shotgun and easily applicable to any nonmodel species (Suchan et al., 2016)—to investigate the spatial and temporal genetic dynamics of the endangered grasshopper *Oedaleus decorus* (Acrididae, Germar 1826). The species is characterized by a large South‐Palearctic distribution, ranging from Macaronesian Islands through the Mediterranean Basin to central Asia (Schmidt & Lilge, 1997). It occupies steppic habitats characterized by dry and warm climates (xero‐thermophilic) and tolerates a relatively narrow temperature range (Ingrisch & Köhler, 1998; Schmidt & Lilge, 1997). The wide distribution of the species might be explained by its ability to fly over long distances (Boczki, 2007; Schmidt & Lilge, 1997). *Oedaleus decorus* is threatened in central Europe because of land‐use changes. For instance, in Switzerland, it is a critically endangered species because of the dramatic loss of steppic habitats that caused a major decrease in population size during the 20th century (Monnerat, Thorens, Walter, & Gonseth, 2007). It is now restricted to two sites in Valais (Gampel and Lower Hérens Valley) and the closest non‐Swiss populations are located in Aosta Valley in Italy and in the Ain department in France (Carron, Fournier, & Marchesi, 1995; Monnerat et al., 2007).

The recent evolutionary history of the species was previously assessed using two mitochondrial loci and 11 microsatellites markers (Kindler, Arlettaz, & Heckel, 2012). However, because of DNA degradation, Kindler et al. (2012) were not able to include samples spanning the whole species distribution area (G. Heckel, unpublished data). In addition, the effects of the recent population decline on the genetic diversity of the species remain unknown. In this study, we first examined the spatial genetic structure of the species with a broader sampling than in the previous work by including additional samples from museum collections—part of them discarded by Kindler et al. (2012). By comparing genetic variation across mitochondrial and nuclear genomes using ultra‐high‐throughput sequencing data, we identified the different genetic lineages of the species across part of its distribution. Second, we investigated the temporal dynamics of genetic diversity and structure among populations by analyzing both current relict and past Swiss populations. Past Swiss populations were represented by museum samples collected during the last century and distributed in the same or neighboring localities to the extant Swiss ones. As a result of the rapid decline of *O. decorus* in Switzerland, a loss of genetic diversity and an increase in population genetic structuring in contemporary samples are expected (Frankham, Ballou, & Briscoe, 2010). Finally, we discuss how the retrieved information can inform about future prospects regarding conservation of *O. decorus* in Switzerland and validate the benefits of incorporating museum samples into genetic analyses.

## 2 MATERIAL AND METHODS

### 2.1 Sample collection and DNA extraction

We gathered 216 fresh and historical specimens of *O. decorus* distributed across the Palearctic (Table S1, Figure S1). Extractions were performed by different researchers, with wetlab protocols thus differing among samples. First, we obtained historical tissue samples from 124 specimens of *O. decorus*, collected between 1884 and 1997, by cutting one middle‐leg. We used an optimized DNA extraction protocol for museum specimens, which are characterized by a highly degraded DNA content (Wandeler et al., 2007). We cleaned all used materials by applying successively bleach, technical EtOH, and UV light. We extracted samples with QIAamp DNA Micro kit (Qiagen, Hombrechtikon, Switzerland) under an UV hood irradiated before each laboratory session to eliminate exogenous DNA. We added DNA yield optimization steps to the protocol supplied by manufacturer: overnight incubation in the lysis buffer was extended to 14 hr and elution was performed with gradual centrifugation in 20.5 μl of Tris–HCl pH 8.5 (10 mmol L^−1^). We eluted the resulting product a second time to increase the amount of isolated DNA. Additionally, muscle tissue from the hind‐legs of 65 fresh specimens collected between 2005 and 2009 and 27 museum specimens (58 of these included in Kindler et al., 2012) were extracted applying a standard phenol–chloroform protocol (Braaker & Heckel, 2009; Kindler et al., 2012). A modified version of the protocol dedicated to degraded DNA was applied to museum specimens extracted by Kindler et al. (2012). For all samples, DNA concentrations were quantified using Quant‐it PicoGreen dsDNA Assay Kit (Thermo Fisher Scientific, Ecublens, Switzerland).

### 2.2 Library preparation and sequencing

We prepared Illumina libraries following the recently developed hyRAD protocol, which allows us to sequence a representative fraction of the genome in a set of samples (Suchan et al., 2016). This method uses hybridization capture and does not strictly rely on PCR amplification. Thus, it is well suited to analyze samples with highly fragmented DNA, such as those isolated from museum specimens. The method consists of three major steps: generation of RAD probes from fresh samples, construction of shotgun libraries from historical and fresh samples of interest, and hybridization capture of the resulting shotgun libraries. Briefly, (i) a double‐stranded RAD library was generated from four fresh DNA samples spread across the species distribution (Hungary, Russia, Spain, and Switzerland, Table S2). Narrow size selection (using a tight mode around 270 bp), removal of adaptor sequences, and biotinylation were applied to the resulting fragments to generate the final probes. (ii) Shotgun libraries were prepared for fresh and historical samples using barcoded adaptors and indexed PCR primers, allowing multiplexing of numerous samples on a single sequencing lane. (iii) Shotgun libraries were captured by hybridization with the biotinylated probes and noncaptured sequences were subsequently washed. Finally, (iv) the captured libraries were reamplified and sequenced on two lanes of Illumina HiSeq 100‐bp paired‐end (Lausanne Genomic Technologies Facility, University of Lausanne, Switzerland).

### 2.3 Illumina data processing

Standard RAD bioinformatics pipelines cannot be performed on target‐enriched libraries because retrieved sequences are not flanked by restriction sites. Consequently, we developed a custom pipeline specifically for hyRAD datasets, derived from the approach described in Suchan et al. (2016; Figure S2).

#### 2.3.1 Demultiplexing and data preparation

Demultiplexing and cleaning of the 100‐bp reads generated by Illumina HiSeq were performed using a customized Perl script based on fastx‐multx and fastq‐clean tools from the *ea‐utilis* package (Aronesty, 2011; Mastretta‐Yanes et al., 2015).

#### 2.3.2 Reference catalogue generation

Eight specimens that generated the largest number of reads after the hyRAD procedure and distributed across the species geographical range (Italy, Spain, Mongolia, and Switzerland) were individually assembled using SOAPdenovo V2.04 with a *k*‐mer size of 31 (Luo et al., 2012). We blasted the generated contigs against GenBank databases for Bacteria, Fungi and technical sequences with a maximum *E*‐value threshold of 0.1 using a custom script (Appendix S1; modified from Nils Arrigo, unpublished data). We considered matching sequences with a minimum length of 80 bp as contaminants, and we subsequently removed them from the reference catalog (see Appendix S2). We assembled the remaining contigs to generate the final catalog using Geneious de novo assembler V9.1.4 (Kearse et al., 2012).

#### 2.3.3 Reads mapping

Before mapping, we processed reads with Trimmomatic V0.3 (Bolger, Lohse, & Usadel, 2014) to remove technical sequences as well as low‐quality nucleotides. We mapped the processed reads with Bowtie 2 V2.2.6 (Langmead & Salzberg, 2012) using the generated catalogue as a reference (see previous section). For historical samples, we evaluated the level of DNA damage by estimating the cytosine deamination probability and the probability of terminating an overhang and we rescaled the base quality scores in accordance with their probability of being damaged using mapDamage V2.0 (Jónsson, Ginolhac, Schubert, Johnson, & Orlando, 2013). We sorted the aligned reads of each individual according to their position on the reference catalogue with BamTools (Barnett, Garrison, Quinlan, Strömberg, & Marth, 2011), and we excluded PCR duplicates by eliminating fragments starting and ending at the same positions using SAMtools V0.1.19 (Li et al., 2009).

For the assessment of temporal genetic changes in Swiss populations, we applied a more stringent method to remove PCR duplicates using the PALEOMIX BAM pipeline (Schubert et al., 2014). Presence of PCR duplicates in the final dataset can induce allele dropout and inflate homozygosity, which can be problematic for population studies requiring fine‐grained comparisons at small spatial or temporal scales (Casbon, Osborne, Brenner, & Lichtenstein, 2011). Briefly, this pipeline consists in trimming adapter sequences of demultiplexed reads, filtering low‐quality reads, and collapsing overlapping paired‐end reads into a single potentially longer sequence. The latter is known to decrease the rate of sequences containing PCR errors and accordingly false SNPs detection (Kircher, 2012). Further steps consist in mapping the reads to the generated catalogue, correcting for postmortem DNA damages in historical samples, and filtering of PCR duplicates.

#### 2.3.4 SNP calling and filtering

We performed SNP calling using FreeBayes V0.9.21‐25, a Bayesian genetic variant detector for short‐read sequencing (Garrison & Marth, 2012). We subsequently applied filters to keep only high‐quality and informative SNPs, as well as informative samples. We removed loci with a Phred quality score below 30 and indels. Then, we kept exclusively biallelic loci with a minor allele count of six (i.e., found at least six times over all samples), present in at least 50% of the samples and with a minimum read depth of six (VCFtools; Danecek et al., 2011). Because high read depth can lead to inflated locus quality score, we excluded loci with a quality score lower than 1/4 of its read depth value (Puritz, Hollenbeck, & Gold, 2014). Finally, we removed potential paralogous loci by applying part of the dDocent filtering pipeline, which detects paralogs based on a coverage three standard deviations higher than the mean (Puritz et al., 2014). We eventually removed samples with more than 80% of missing data from the final dataset. We determined final mean read depths per site and per individual with VCFtools (Danecek et al., 2011), and we assessed the effect of initial DNA concentration and sampling year on genotyping success using a binomial generalized linear model. To identify whether historical and modern data performed similarly in terms of output data, we compared mean read length as well as percentage of missing data between historical and modern samples using a Mann–Whitney *U* test. Finally, we generated input files for further analyses from the final VCF SNPs matrix using PGDSpider V2.0.9.0 (Lischer & Excoffier, 2012).

#### 2.3.5 Mitochondrial data

Because (most but) not all sequences nonhomologous to the probes are filtered out during the enrichment step of hyRAD, it becomes possible to retrieve genes present in multiple copies, which are found at high coverage in the DNA samples. This is notably the case of the mitochondrial genome, which we partly retrieved in 40 specimens with the highest number of reads and spanning the species distribution (Table 1). We mapped the cleaned reads against the mitochondrial genome of the closely related species *Oedaleus asiaticus* (GenBank accession number EU513374.1) with BWA V0.7.12 (Li & Durbin, 2009). We sorted the generated bam files with SAMtools V0.1.19 (Li et al., 2009) and removed duplicates with Picard V2.4.1 (http://broadinstitute.github.io/picard/). We realigned the subsequent bam files with GenomeAnalysisTK V2.8 (DePristo et al., 2011), called SNPs with GenomeAnalysisTK *UnifiedGenotyper*, and performed diploid phasing with GenomeAnalysisTK *ReadBackedPhasing*. We identified and removed potential NUMTs with Odintifier (Castruita, Mendoza, Barnett, Wales, & Gilbert, 2015). We built consensus sequences with the resulting bam files in Geneious V9.1.4 (Kearse et al., 2012) and generated multiple alignments with MAFFT V7.222 (Katoh, Misawa, Kuma, & Miyata, 2002). We used BMGE (Block Mapping and Gathering with Entropy) V1.12 (Criscuolo & Gribaldo, 2010) to select phylogenetic informative regions, removed sites with more than 50% of gaps, and discarded samples with more than 90% of missing data.

**Table 1:** Populations of *O. decorus* included either in the spatial or in the temporal genetic structure analyses

Population	Location	Country	Sampling year	hyRAD	mtDNA
***Spatial analysis***					
Algeria	Djelfa	Algeria	1938	2	0
Croatia	Krk	Croatia	2005	2	1
Corse	Piana, Capo Rossa, Corse	France	2006	2	0
Crau	St‐Martin de Crau	France	2009	2	1
Lyon	Lyon, Valbonne	France	2008	1	1
Montolivet	Montolivet	France	1954	2	0
Sisteron	Sisteron	France	2005	1	0
Chelmos	Mt. Chelmos, nr. Kalavryta	Greece	1938	1	0
Diakopto	Diakopto	Greece	1938	2	0
Fülöphaza	Fülöphaza	Hungary	1977	0	1
Caulonia	Caulonia, Monte Gremi	Italy	1948	1	0
Certaro	Certaro, Calabria	Italy	1948	1	1
Cogne	Valle di Cogne, Aosta Valley	Italy	2005	2	1
Lampedusa	Lampedusa, Capo Peneto	Italy	1969	1	0
Popoli	Capo Pescara, Popoli	Italy	1992	1	1
Randazzo	Randazzo, Etna	Italy	1969	2	0
Sicily	Rocca di Novara, Sicily	Italy	1967	2	1
Susa	Susa	Italy	2009	2	0
Tremiti	Isola Tremiti, San Nicola	Italy	1954	1	0
Udine	Udine, Magredi di Cordenons	Italy	2009	2	1
Dashinchilen	Dashinchilen	Mongolia	1968	2	1
Zogt‐Ovoo	Zogt‐Ovoo	Mongolia	1967	2	1
Fez	Fez	Morocco	1968	0	2
Areiro	Pico de Areiro, Madeira	Portugal	1980	2	0
Boca	Boca dos Corgos, Madeira	Portugal	1978	2	1
Encumenda	Encumenda, Madeira	Portugal	1954	1	0
Estrella	Serra da Estrella	Portugal	1933	1	1
Mangaulde	Mangaulde	Portugal	1969	1	0
Paul	Paul da Serra, Madeira	Portugal	1964	1	0
Tapa da Ajuda	Tapa da Ajuda	Portugal	1934	1	0
Kurgan	Kurgan	Russia	2009	1	0
Canary	Tenerife, Canary Is.	Spain	1966	1	0
Capilieira	Sierra Nevada, Capileira	Spain	2007	2	0
Granada	Granada, Puerto de la Mora	Spain	1965	1	0
Guadarrama	Sierra de Guadarrama	Spain	1958	2	1
Ausserberg	Ausserberg	Switzerland	1963	1	0
Finges	Finges	Switzerland	1939–1954	14	3
Follatères	Follatères	Switzerland	1931	1	0
Gampel	Gampel	Switzerland	2005	18	2
Lower Hérens Valley	Lower Hérens Valley	Switzerland	1908–2005	23	4
Saas	Saas	Switzerland	1938	1	1
Sierre	Sierre	Switzerland	1908–1941	5	2
St‐Niklaus	St‐Niklaus	Switzerland	1927	1	1
Malatya	Malatya	Turkey	1930	1	1
Mugla	Mugla Vilayet	Turkey	1947	1	1
Niksar	Niksar	Turkey	1959	1	0
Sivrihisar	Sivrihisar	Turkey	1969	1	0
Urfa	Urfa	Turkey	1931	1	0
***Temporal analysis***					
Ausserberg	Ausserberg	Switzerland	1963	1	N/A
Finges 1940	Finges	Switzerland	1939	5	N/A
Finges 1950	Finges	Switzerland	1949	7	N/A
Finges 1954	Finges	Switzerland	1954	1	N/A
Follatères	Follatères	Switzerland	1931	1	N/A
Gampel 2005	Gampel	Switzerland	2005	18	N/A
Lower Hérens Valley 1908	Lower Hérens Valley	Switzerland	1908	1	N/A
Lower Hérens Valley 1940	Lower Hérens Valley	Switzerland	1940–1941	4	N/A
Lower Hérens Valley 1997	Lower Hérens Valley	Switzerland	1997	3	N/A
Lower Hérens Valley 2005	Lower Hérens Valley	Switzerland	2005	15	N/A
Saas	Saas	Switzerland	1938	1	N/A
Sierre 1908	Sierre	Switzerland	1908	1	N/A
Sierre 1940	Sierre	Switzerland	1941	4	N/A

Given are population identifier, sampling site (location, country), sampling year, and number of samples used, respectively, for hyRAD‐based SNPs and mtDNA analyses (hyRAD, mtDNA). Samples used for the temporal analysis are embedded into those used for the spatial analysis.

### 2.4 Spatial analysis

We used a combination of fresh samples and samples collected during the past decades for the spatial analysis. Thus, we assumed that local genetic differences accumulated over the last century are negligible in comparison with the spatial genetic variations accumulated over the Quaternary evolutionary history of *O. decorus* for reconstructing continent‐level phylogeographic patterns.

#### 2.4.1 Spatial genetic structure identified from SNP data

We inferred population structure and individual assignment using fastSTRUCTURE V1.0, a STRUCTURE‐like algorithm dedicated to large SNP genotype data (Raj, Stephens, & Pritchard, 2014). We performed the analyses assuming *k* numbers of groups ranging from 1 to 10 and a simple prior (i.e., a flat beta‐prior over population‐specific allele frequencies at each locus), which is appropriate to study populations isolated from each other (Raj et al., 2014). We determined the most likely *k* number of clusters with the program chooseK.py included in the fastSTRUCTURE package. We performed individual visualizations of admixture proportions using DISTRUCT V1.1 (Rosenberg, 2004), and we represented the geographical distribution of the genetic clusters using Quantum GIS V2.4.0 (Quantum GIS Development Team, 2015).

#### 2.4.2 Spatial genetic structure identified from mitochondrial data

We used PhyML V3.1 (Guindon et al., 2010) to perform maximum‐likelihood phylogenetic reconstruction on the retrieved mitochondrial sequences (9,892 bp after the application of BMGE and removal of sites with low sample representation) with a TN93 + G + I + F model (i.e., the best model as inferred by PhyML V3.1). We used two samples of *Oedaleus decorus asiaticus* from Mongolia as outgroups. We assessed topology robustness with 1,000 bootstrap replicates and, in addition, calculated the support for each branch using the aBayes algorithm, a Bayesian‐like transformation of approximate likelihood ratio test (Anisimova, Gil, Dufayard, Dessimoz, & Gascuel, 2011), as implemented in PhyML. We visualized and annotated the resulting tree in Geneious V9.1.4 (Kearse et al., 2012).

### 2.5 Temporal analysis

We based the temporal analysis only on Swiss samples. First, we computed the differences in individual heterozygosities among all past and extant samples. Then, to test the expectation of loss of genetic diversity associated with the regional extinction of populations, we focused on the six Swiss populations (Finges 1940, Finges 1950, Sierre 1940, Lower Hérens Valley 1940 and 2005, Gampel 2005, see Table 1) for which the number of individuals that passed all filtering stages was equal to or higher than four. Populations were considered different if collected in a different location, or in the same location but across a time interval of at least 10 years.

Sample size in each historical population was too small (*n *≤* *7) to detect deviation from Hardy–Weinberg expectations (HWE). Thus, we performed exact tests for deviation from HWE only within the two extant populations using 1,000 permutations with the Monte Carlo procedure implemented in the R‐package pegas (Paradis, 2010). In order to correct for multiple testing, we applied the false discovery rate (FDR) method (Benjamini & Hochberg, 1995) and computed the *q*‐value for each test using the R‐package qvalue (Storey, Bass, Dabney, & Robinson, 2015). Loci with significant *q*‐value (*q *<* *0.05) were excluded from further analyses.

Observed heterozygosity (*H*_obs_) was calculated for each individual using the R‐package hierfstat (Goudet, 2005). Levels of individual observed heterozygosity between historical and contemporary samples were compared using a Mann–Whitney *U* test.

For the population‐level analysis, we removed SNPs with a fraction of missing data higher than 70% within each population. Observed heterozygosity (*H*_obs_), expected heterozygosity (*H*_exp_, also known as genetic diversity; Goudet, 2005), and rarefied allelic richness (*A*_R_) were calculated using hierfstat (Goudet, 2005). We tested for pairwise differences in mean *H*_obs_, *H*_exp_, and *A*_R_ between each historical and contemporary population using 10,000 permutations and *p*‐values were adjusted for multiple comparisons using the FDR method. Number of private alleles between contemporary populations and between historical and contemporary populations of the same location was determined with ADZE V1.0 (Szpiech, Jakobsson, & Rosenberg, 2008), and their frequency was calculated with VCFtools (Danecek et al., 2011). Population genetic structure was characterized with pairwise estimates of Weir and Cockerham fixation index (*F*_ST_) to control for difference in population size using adegenet (Jombart & Ahmed, 2011). Significance of pairwise *F*_ST_ was tested with 10,000 permutations, and *p*‐values were corrected for multiple comparisons with the FDR method.

Finally, we examined the demographic dynamics in the sole population with a sufficient number of samples for at least two time points, that is, Finges in 1940 and 1950. More specifically, we determined whether the population decline was linear or exponential over time. We tested 30 scenarios of population decline. Each scenario is characterized by an initial and a final population size as well as a type of decline (one linear and two exponentials; see Table S3). For each scenario, the minor allele frequencies were calculated for both Finges 1940 and 1950 populations using the R‐package vcfR (Knaus & Grünwald, 2017). Then, starting from the minor allele frequency distribution of Finges 1940, we simulated a new minor allele frequency distribution based on the described scenarios of population decline over ten generations (see Appendix S3 for script). The distribution of simulated minor allele frequency was then compared to the observed distribution in Finges 1950 using a bootstrapped version of the Kolmogorov–Smirnov test implemented in the matching R‐package (Sekhon, 2011), and *p*‐values were corrected for multiple comparisons with the FDR method. We performed all statistical analyses in R V3.3.2 (R Core Team, 2016).

## 3 RESULTS

HyRAD libraries yielded a total of 196,158,914 raw reads, of which 80.3% were retained after quality filtering. Of the 216 samples, 119 (ca. 55%; 68 historical and 51 fresh; Table 1) were successfully sequenced with quality checks above all thresholds. DNA concentration at extraction had a significant effect on sequencing success coded as a binomial variable (concentration: χ^2^ = 33.4, *df* = 1, *p *=* *8e−9; Figure S3). No significant difference in read length (*W* = 4190.5, *p *=* *.14; Figure S4) and proportion of missing data (*W* = 1818.5, *p *=* *.72; Figure S5) was found between modern and historical samples. Initial average number of contigs in the eight samples used to create the reference catalog was 376,455. Of them, 42,822 were longer than 80 bp. The final reference catalog was composed of 33,023 contigs after removal of contaminant sequences and merging of all samples.

### 3.1 Spatial genetic structure

#### 3.1.1 Dataset description

After all filtering steps, we retrieved a final number of 1,165 SNPs spanning 578 loci (see Table S3 for details of filtering steps). The matrix completeness was of 60% and the average read depth for each SNP was 12 (Figure S6A). An average of 698 SNPs characterized each sample (Figure S6B). For the mitochondrial DNA analysis, after the application of BMGE, a 9,892‐bp‐long consensus sequence—which represents 60% of the complete mitogenome—was retrieved for 31 of the 40 samples selected and the matrix completeness was 69%.

#### 3.1.2 Spatial genetic structure

**Figure 1:**
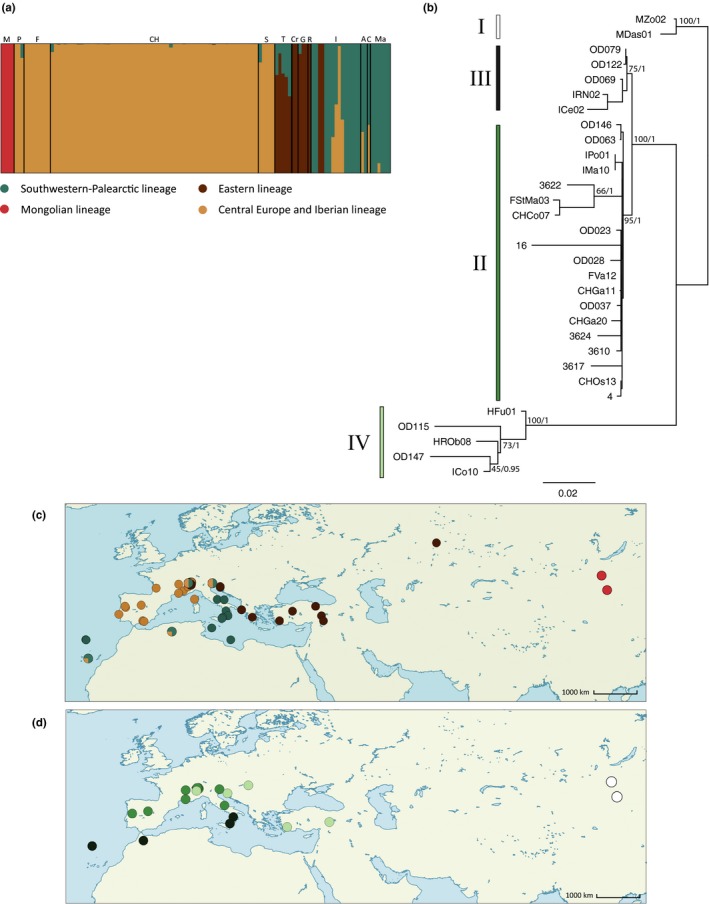
(a) Individual Bayesian cluster analysis of hyRAD SNP data using fastSTRUCTURE (Raj et al., 2014) based on 1,165 SNPs and assuming four genetic clusters; the letters stand for the populations of origin of each sample. M: Mongolia (corresponding to *Oedaleus decorus asiaticus* samples); P: Portugal; F: France; CH: Switzerland; S: Spain; T: Turkey; Cr: Croatia; G: Greece; R: Russia; I: Italy; A: Algeria; C: Canary Islands; Ma: Madeira. (b) Maximum‐likelihood phylogenetic tree obtained with PhyML (Guindon & Gascuel, 2003) based on a reduced panel of 31 samples using 9,892‐bp‐long mtDNA sequences. Bootstrap support values for the phylogeny generated using 1,000 resampled datasets and aBayes nodes support are, respectively, indicated in first and second positions next to the major nodes. Roman numerals stand for the four main phylogenetic clades. (c) Spatial representation of the SNP‐based genetic structure illustrated in (a), with the proportion of each population assigned to each cluster shown with different colors as a pie chart. (d) Spatial representation of the four mtDNA clades shown in (b)

The genetic structure inferred with fastSTRUCTURE from the nuclear SNP data was best explained by four clusters (*k *=* *4, Figure 1a,c), hereafter referred to as Mongolian, central European/Iberian, Southwestern‐Palearctic, and Eastern lineages. The first group consisted of individuals from Mongolia, which correspond to the subspecies *O. decorus asiaticus*. The central European/Iberian cluster included all individuals from Switzerland, France, Spain, and Portugal. The Southwestern‐Palearctic group encompassed individuals from the southern part of the species range (i.e., Algeria, Madeira, Canary Islands, Lampedusa, Southern Italy). The Eastern cluster was composed of specimens originating from the Eastern part of the species range (i.e., Greece, Turkey, Croatia, Russia) as well as individuals coming from the Aosta Valley in northern Italy. Finally, specimens originating from northeastern and northwestern Italy were assigned to both southern and central European/Iberian lineages. Introgression of the southern lineage was also found in half of the individuals assigned to the Eastern lineage. The maximum‐likelihood phylogeny based on mitochondrial DNA was congruent with the structure of those four clusters (Figure 1b,d).

### 3.2 Temporal genetic structure

#### 3.2.1 Dataset description

A total of 278,634 polymorphic sites were retrieved by SNP calling in the Swiss samples. After filtering, we retrieved 1,444 SNPs spanning 593 loci (see details of the filtering step in Table S4). We obtained genotype information for 62 samples (33 fresh, 29 historical; Table 1). SNP coverage was 16.6 reads on average (17.6 for historical samples and 15.6 for contemporary samples; Figure S7A), and the matrix completeness was 65.3%. An average of 1,096 SNPs characterized each sample (1,062 for historical samples and 1,130 for contemporary samples; Figure S7B).

For the population‐level analysis, we considered only populations with at least four samples and thus reduced the dataset to 55 samples (34 fresh, 21 historical; Table 1). Of the 1,444 SNPs previously retrieved, only a reduced fraction of 328 SNPs had less than 70% of missing data within each population and were subsequently used.

#### 3.2.2 Population genetic structure

**Figure 2:**
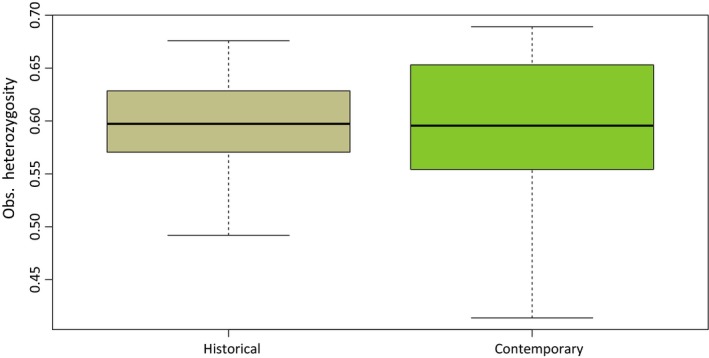
Distribution of observed heterozygosity at the individual level from historical (i.e., before 1955; *n *= 29) and contemporary (*n *=* *33) Swiss samples. Top and bottom of the box, respectively, represent the upper and the lower quartile, and the bold line corresponds to the median (both groups are not significantly different in terms of observed heterozygosity; *W* = 467.5, *p *=* *.88)

**Figure 3:**
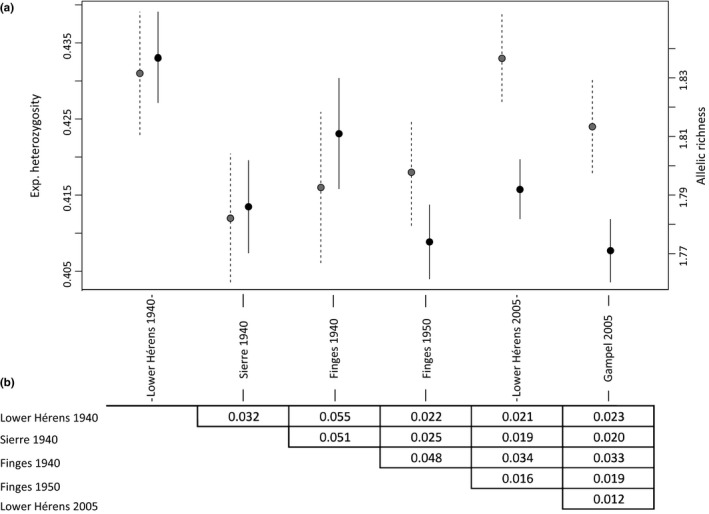
(a) Expected heterozygosity (gray dots and dashed error bars) and allelic richness (black dots and solid error bars) averaged over 328 SNPs in historical (1940 and 1950) and contemporary (2005) *O. decorus* populations. Error bars represent standard error. (b) Pairwise genetic distance (*F*_ST_) among six contemporary and historical populations. None of the values are significant (α = 0.05) after standard false discovery rate correction (Benjamini & Hochberg, 1995)

**Figure 4:**
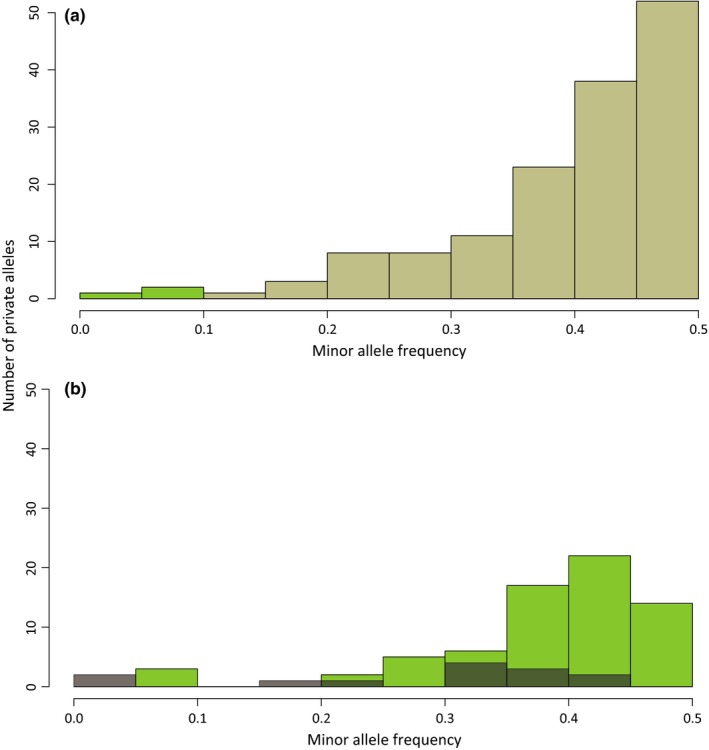
Distribution of minor allele frequency based on the private alleles identified (a) between Lower Hérens Valley 1940 (brown) and Lower Hérens Valley 2005 (green) and (b) between Lower Hérens Valley 2005 (green) and Gampel 2005 (gray)

Mean observed individual heterozygosity did not differ significantly between historical and contemporary samples (*W* = 467.5, *p *=* *.88; Figure 2). For the population‐level analysis, observed heterozygosity was higher than expected heterozygosity in all populations (Table 2). Pairwise differences for expected heterozygosity and observed heterozygosity were not significant after FDR correction for multiple testing whatever pair of populations considered (*p *<* *.05; Table 2). Allelic richness comparisons among populations are given in Table 2. Pairwise *F*_ST_ estimates were not significant between each population after FDR correction whatever pair of populations considered (Figure 3b). Still, the smallest genetic distance was found between the two contemporary populations (i.e., Lower Hérens Valley 2005 and Gampel 2005; Figure 3b). For a total of 328 alleles, comparison of Lower Hérens Valley 2005 (*n *=* *15) and Lower Hérens Valley 1940 (*n *=* *4) showed three private alleles with low frequency in Lower Hérens Valley 2005 and 144 private alleles in Lower Hérens Valley 1940 (Figure 4a). When comparing Gampel 2005 (*n *=* *18) and Lower Hérens Valley 2005 (*n *=* *15), we detected 15 private alleles in Gampel 2005 and 71 private alleles in Lower Hérens Valley 2005 (Figure 4b).

**Table 2:** Summary statistics for each population

Population	*n*	*H*_obs_	*H*_exp_	*A*_r_
Finges 1940	5	0.615^a^	0.416^a^	1.811^ab^
Finges 1950	7	0.680^a^	0.418^a^	1.774^b^
Gampel 2005	18	0.649^a^	0.424^a^	1.771^b^
Lower Hérens Valley 1940	4	0.669^a^	0.431^a^	1.837^a^
Lower Hérens Valley 2005	15	0.678^a^	0.433^a^	1.792^ab^
Sierre 1940	4	0.615^a^	0.412^a^	1.786^ab^

Given are the sample size (*n*), observed heterozygosity (*H*_obs_), expected heterozygosity (*H*_exp_), and rarefied allelic richness (*A*_r_). Different letters indicate significant mean pairwise differences after adjustment for multiple testing with the Benjamini and Hochberg (1995) false discovery rate method (*p *<* *.05).

Finally, the 30 scenarios tested to simulate the population decline in Finges between 1940 and 1950 all generated minor allelic frequency distributions significantly different from the observed values (*p *<* *.05; Table S3), indicating that none of the scenarios tested fitted our data, likely revealing a lack of statistical power in our dataset.

## 4 DISCUSSION

The aim of this study was to combine genetic data generated from museum specimens and modern samples to reconstruct the spatial genetic structure of *O. decorus* across its distribution range and to uncover the changes in genetic diversity over the past decades at the Swiss scale. We used hyRAD, a recently developed technique to retrieve genome‐wide sequences in nonmodel organisms and applicable to any kind of samples, including those characterized by fragmented DNA. At the spatial level, we analyzed mitochondrial and (mostly nuclear) SNP data and highlighted four lineages, which produced spatial genetic clustering at the scale of the western Palearctic that were generally consistent between both approaches. At the temporal level, we identified a decrease in allelic richness in contemporary populations. Our study demonstrates the utility of DNA isolated from museum specimens when associated with hybridization capture methods, to investigate genetic changes related to population decline and to achieve a wide geographical coverage of samples to establish the spatial genetic structure of nonmodel organisms.

### 4.1 Western‐Palearctic‐wide genetic structure

Mitochondrial and nuclear DNA genetic structuring of *O. decorus* was consistent. Four geographically structured groups were recognized (Figure 1). Such congruence between mitochondrial and nuclear genetic structuring—with the exception of one population from central Italy—contrasts with patterns of cytonuclear discordance previously found in the population of the Valle d'Aosta (Italy)—both historical introgression of mtDNA and sex‐specific gene flow were suggested as an explanation (Kindler et al., 2012)—a discrepancy possibly due to the lower number of specimens analyzed for this locality in the present study.

#### 4.1.1 Potential glacial refugia and postglacial range expansions

The observed genetic structuring of *O. decorus* across part of its distribution range (Figure 1) can be explained by the survival of the species in three Quaternary European (or western Palearctic) glacial refugia that remained isolated during the last glacial maximum (LGM). Similar patterns were found in various temperate European species (Taberlet et al., 1998) and are suggested to be caused by range shifts toward climatically suitable refugia during the LGM followed by postglacial recolonization of new regions (Hewitt, 1999). The Eastern and Iberian/central Europe lineages of *O. decorus* probably recolonized Europe from classical refugia after the LGM (i.e., respectively, the southern Balkans and the Iberian Peninsula). However, two hypotheses can account for the postglacial expansion of the Southwestern‐Palearctic lineage: (i) recolonization of Europe from southern Italy or (ii) from northern Africa. Italy is generally considered as a major refugium in southern Europe (Hewitt, 1999; Ruedi et al., 2008), but most biogeographical studies often overlooked the importance of North Africa as a source for postglacial colonization of Europe (Husemann, Schmitt, Zachos, Ulrich, & Habel, 2014b). Several phylogeographical studies highlighted that European lineages are nested within African clades, suggesting that colonization from North Africa toward Europe is relatively common and reinforcing the role of North Africa as glacial refugium (see Husemann et al., 2014b for a review). Postglacial recolonization from Maghreb was suggested in different species, such as *Apis mellifera* (Franck, Garnery, Solignac, & Cornuet, 1998) or *Melitaea cinxia* (Wahlberg & Saccheri, 2007). Combining this information with the high dispersal ability of *O. decorus* suggests that North Africa could be a possible refugium for the species. Furthermore, our results indicate that North Africa may also have acted as a source for the colonization of the Canary Islands and Madeira. Most taxa colonized these islands from Africa or from the Iberian Peninsula using airstreams (Díaz‐Pérez, Sequeira, Santos‐Guerra, & Catalán, 2008; Hochkirch & Goerzig, 2009). For example, passive wind dispersal from North Africa was hypothesized as the main mean of colonization of Canary Islands for the majority of the fully winged grasshopper species of the genus *Sphingonotus* (Husemann, Deppermann, & Hochkirch, 2014a).

#### 4.1.2 The Alps as a cross‐roads of genetic lineages

Three genetic lineages meet in the Alpine and peri‐Alpine region, suggesting that the Alps act as an important cross‐roads for *O. decorus*. The Alpine barrier is a known major suture zone for many species, where different evolutionary lineages encounter (Taberlet et al., 1998; Triponez, Arrigo, Pellissier, Schatz, & Alvarez, 2013). Our SNP‐based results suggest admixture between lineages in two Italian populations in the peri‐Alpine region (Figure 1a,c). Such a process is known to increase genetic diversity because individuals originating from distinct glacial refugia will carry different alleles (Hewitt, 2004). In addition, cytonuclear discordance was found in one population further south in Italy, also suggesting introgression between lineages associated with different refugia.

### 4.2 Change in genetic diversity through time at the Swiss scale

We detected significant differences among Swiss populations in allelic richness (Figure 3a), a measure known to be more sensitive to population decrease compared to expected heterozygosity (Cornuet & Luikart, 1996), which remained stable over time. Such a pattern was previously highlighted in threatened species using one single time point (e.g., Pinsky & Palumbi, 2014; but see Ugelvig, Nielsen, Boomsma, & Nash, 2011) and is due to the rapid loss of rare alleles during population decline, which in turn will have only a limited effect on heterozygosity (Hedrick, Brussard, Allendorf, Beardmore, & Orzack, 1986). In the case of *O. decorus*, the observed pattern might be explained, at least for populations in Finges (which was sampled twice, in 1940 and 1950) and Lower Hérens Valley (which was sampled twice, in 1940 and 2005), by an increase in the bottleneck magnitude over time. Although we could not identify a clear‐cut demographic scenario for the Finges population—the population with the largest number of collected samples in our study (Table S3)—the private alleles pattern found in the Lower Hérens Valley population suggests substantial genetic erosion in only 65 years (144 vs. 3 private alleles in 1940 and 2005, respectively). Whereas the high number of biallelic loci analyzed here should compensate for the absence of multiallelic markers to estimate allelic richness (Ryynänen, Tonteri, Vasemägi, & Primmer, 2007), we acknowledge the present dataset is limited in terms of sample size, and alternatives to the latter hypothesis might still be at work. Whereas several methods have been developed to unravel the evolutionary history of species at a large temporal scale when sampling is limited (e.g., PSMC, Li & Durbin, 2011; MSMC Schiffels & Durbin, 2014), those cannot be applied at shorter temporal scales, for which larger numbers of individuals per location and time point should be sampled to accurately compute genetic parameter estimates (Buerkle & Gompert, 2013).

With low mutation rate markers such as SNPs, *F*_ST_ is usually assessed more reliably compared to other estimators such as *G*′_ST_ of Jost's D (Whitlock, 2011). However, no pairwise differentiation between populations was significant, which might be explained here as well by a lack of statistical power related to our small sample size. Thus, *F*_ST_ values should be interpreted with caution. Still, it is interesting to note that the lowest differentiation was found between the two contemporary populations of Lower Hérens Valley and Gampel (Figure 3b). This contrasts with the usual view that a decrease in population size will lead to population fragmentation and consequently, to a higher differentiation (Frankham, 2005). Furthermore, a low number of private alleles were found in Gampel when compared to Lower Hérens Valley (Figure 4b). While further analyses are necessary to establish the amount of gene flow between these populations, our results are compatible with the presence of *O. decorus* in Gampel prior to a population enrichment by natural dispersal or through a possible recent deliberate human translocation of individuals from Lower Hérens Valley, as suspected by experienced entomologists who had never recorded that species at this site before the early 21st century (R. Arlettaz, unpublished data; see Figure S8).

### 4.3 Conservation implications

In this study, we focused on genetic variation patterns of *O. decorus*. However, genetic variation is only one of various factors linked with population viability. Population dynamics (e.g., number of individuals, growth rate, variation in demographic parameters) and environmental effects (e.g., interactions with other species, habitat quality and quantity) are known to greatly influence species extinction risk (Mace & Lande, 1991). Especially, various studies suggested that orthopteran species are highly sensitive to landscape alterations (Gauffre et al., 2015; Keller et al., 2013; Ortego, Aguirre, Noguerales, & Cordero, 2015). *Oedaleus decorus* heavily relies on open bare ground habitat, but as a result of lower grazing pressure, vineyard and urbanization expansion, such habitats are becoming extremely scarce in the modern Swiss landscapes (Monnerat et al., 2007). The species is known from only two extant locations in Switzerland, which leads to an increased extinction risk through stochastic events (e.g., diseases, climate). Thus, we support the current conservation management, which aims to establish new populations of *O. decorus* in Valais. We further propose to use individuals for the translocations either from Lower Hérens Valley, as it is the one showing the highest levels of allelic richness, or from both Lower Hérens Valley and Gampel. Indeed, private alleles present at high frequency in Gampel and Lower Hérens Valley suggest that both populations should be considered as separate genetic units, even though differentiation was not significant. Finally, current Valais populations do not show large amounts of genetic impoverishment despite their current isolation, suggesting either resilience of *O. decorus* to fragmentation in these two specific locations or, less likely, recurrent immigration of individuals.

#### 4.3.1 Application of hyRAD: future prospects and challenges in museum genomics

In this study, we took advantage of the availability of museum specimens from different locations and time points to provide an insight into the genetic dynamics of *O. decorus* at both spatial and temporal scales. By successfully applying hyRAD to 119 specimens, we retrieved more than a thousand of SNPs, a major improvement compared to previous studies using museum specimens (e.g., Kindler et al., 2012; Spurgin et al., 2014), confirming the recent findings of Linck, Hanna, Sellas, and Dumbacher (2017), who successfully tested hyRAD on bird samples.

Numerous studies advocate the benefits of combining museum and contemporary samples to test ecological and evolutionary hypotheses (Spurgin et al., 2014; Tsangaras & Greenwood, 2012; Wójcik, Kawałko, Marková, Searle, & Kotlík, 2010). However, working with historical samples is challenging and is usually associated with several pitfalls both at the sampling and at molecular levels. Allelic dropout is a recurrently reported concern when dealing with low concentrations of DNA, such as DNA extracted from museum specimens (Taberlet et al., 1996; Wandeler et al., 2007). Allelic dropout is a stochastic event occurring when only one of the two alleles present at a heterozygote locus is amplified (Taberlet et al., 1996). Consequently, multiple copies of a single initial DNA template are sequenced (i.e., PCR duplicates), which will systematically inflate the level of homozygosity (Casbon et al., 2011; Figure S9). This issue is more important when comparing past and present genetic diversity at a narrow spatial and temporal scale, because subtle differences in genetic diversity are expected. In contrast, when historical specimens are combined with contemporary samples to increase the geographical coverage of broad‐scale studies, the noise generated by allelic dropout and resulting PCR duplicates is not problematic, because of the usually random distribution of the historical samples across localities as well as of the large genetic divergence expected among lineages that diverged following Quaternary climatic oscillations. In the short term, sensitive bioinformatic steps such as catalogue generation (e.g., assembler type and k‐mer value), SNPs calling, and paralogs filtering may deserve further optimizations, especially for species with large genome, such as the focal species of our study. In addition, population genetics analyses might be limited by the number of museum samples available through time and space. It is well known that the number of samples—as well as the number of alleles and loci—influences statistical power of genetic analyses (Ryman et al., 2006). Investigating the number of samples available in museums beforehand would allow researchers to determine which questions could be answered as well as their associated potential statistical power. In the analyses presented here, we acknowledge that sample sizes are rather low and therefore interpretation of our conclusions should be taken with caution. We thus advocate that natural history museums from all around the world should produce a joint effort to share a general database including information about origins and ages of all available specimens; such a resource would be highly valuable for the future of museum genomics.

## CONFLICT OF INTEREST

None declared.

## AUTHOR CONTRIBUTIONS

S.S., R.A., and N.A. designed the project. G.H. provided DNA extractions of all fresh samples and part of historical samples. S.S. and N.A. coordinated analyses. S.S. performed laboratory work with the help of C.P. and N.A. M.P. contributed to script development. S.S. analyzed the data with the help of S.N. S.S. drafted the manuscript. All authors revised and approved the manuscript.

## DATA ACCESSIBILITY

Raw reads, mitochondrial DNA alignment as well as the VCF datasets used for the analyses are available in ZENODO (https://doi.org/10.5281/zenodo.1037125).
