# Supplementary material for: Spatial and temporal genetic dynamics of the grasshopper Oedaleus decorus revealed by museum genomics
Source: Ecol Evol. 2017 Dec 29;8(3):1480–95. doi: 10.1002/ece3.3699 (PMC5792620; doi:10.1002/ece3.3699)
Supplement: Supplementary file 1 [file ECE3-8-1480-s001.pdf]

SUPPORTING INFORMATION

The Supporting information includes three Supplementary tables, nine Supplementary figures and three Appendices.

Table S1 Sampling locations of all *O. decorus* samples used in this study across the species distribution range in Europe, northern Africa and central Asia.

Table S2 Samples used for the generation of the probes in the hyRAD protocol.

Table S3 Scenarios of population decline tested for the Finges population between 1940 and 1950 as well as the associated statistic for the comparison between the simulated and observed minor allele frequency distribution.

Table S4 Number of polymorphic sites after each filtering step for both spatial and temporal dataset.

Figure S1 Spatial distribution of all available *O. decorus* samples across its distribution range in Europe, northern Africa and central Asia.

Figure S2 Custom bioinformatics pipeline to retrieve SNPs from hyRAD data.

Figure S3 Success of sequencing according to the DNA concentration of each sample.

Figure S4 Distribution of read length for all historical (n = 68) and contemporary (n = 51) samples.

Figure S5 Distribution of the fraction of missing data for all sequenced historical (n = 68; brown) and contemporary (n = 51; green) samples.

Figure S6 Distribution of mean read depth per site and informative SNPs per sample for the 119 samples originating throughout the species geographical range. The dark line indicates the mean.

Figure S7 Distribution of mean read depth per site and informative SNPs per sample for the 62 Swiss samples. The dark line indicates the mean.

Figure S8 Timeframe illustrating the Swiss *O. decorus* populations used in this study.

Figure S9 Distribution of observed heterozygosity at the individual level both in historical and contemporary samples when PCR duplicates are not removed carefully.

Appendix S1 Blast search against GenBank and matching sequences extraction.

Appendix S2 Removing contaminant sequences from reference catalog**.**

Appendix S3 Simulation of population decline in Finges population.

SUPPLEMENTARY TABLES

Table S1 Sampling locations of all *O. decorus* samples used in this study across the species distribution range in Europe, northern Africa and central Asia. Given are sample label, population name as used for spatial and temporal analyses (cross-referenced with Table 1), sampling site (country, location, and coordinates), collection year, DNA concentration after extraction (in ng/µl) and initials of the museum's curators and scientists who provided the specimens (i.e. Source). Museum specimens are indicated with stars and successfully sequenced samples used for further analysis are in bold. Sources are as follows: GH (Gerald Heckel, University of Bern), HB (Hannes Baur, Natural History Museum Bern), AF (Anne Freitag, Zoological Museum Lausanne), RE (Rod Eastwood, ETH Entomological Collection), DB (Daniel Burckhardt, Natural History Museum Basel), PS (Peter Schwendinger, Natural History Museum Geneva), GB (George Beccaloni, Natural History Museum London), BO (Barabara Oberholzer, Natural History Museum Zurich), RA (Raphaël Arlettaz, University of Bern).

Label	Population name		Country	Location	Latitude	Longitude	Year	Conc.	Source
	Spatial analysis	Temporal analysis							
**OD096***	Algeria	-	Algeria	Djelfa	34.67517	3.26842	1938	34.33	GB
**OD099***	Algeria	-	Algeria	Djelfa	34.67517	3.26842	1938	17.80	GB
BGPe01*	-	-	Bulgaria	Petrich	41.39811	23.20669	1960	1.85	GH
HROb05	-	-	Croatia	Krk, Obzovo	45.02790	14.57521	2005	6.85	GH
**HROb08**	Croatia	-	Croatia	Krk, Obzovo	45.02790	14.57521	2005	11.60	GH
HRVo01	-	-	Croatia	Krk, Obzovo	45.02790	14.57521	2005	24.51	GH
**HRVo10**	Croatia	-	Croatia	Krk, Voz	45.23187	14.57956	2005	28.73	GH
FVa03	-	-	France	Ain, Lyon, Valbonne	45.76404	4.83566	2008	3.95	GH
**FVa12**	Lyon	-	France	Ain, Lyon, Valbonne	45.76404	4.83566	2008	3.69	GH
009*	-	-	France	Lavandou	43.13804	6.36844	1952	0.83	HB
**010***	Montolivet	-	France	Montolivet	44.84967	-0.45024	1954	0.83	HB
**011***	Montolivet	-	France	Montolivet	44.84967	-0.45024	1954	1.44	HB
**FPi05**	Corse	-	France	Piana, Capo Rossa, Corse	42.23639	8.54111	2006	7.64	GH
**FPi14**	Corse	-	France	Piana, Capo Rossa, Corse	42.23639	8.54111	2006	51.40	RA
FSi01	-	-	France	Sisteron	44.19471	5.94319	2005	8.96	RA
**FSi02**	Sisteron	-	France	Sisteron	44.19471	5.94319	2005	64.05	RA
**FStMa02**	Crau	-	France	St-Martin de Crau	43.63863	4.81211	2009	96.74	RA
**FStMa03**	Crau	-	France	St-Martin de Crau	43.63863	4.81211	2009	105.18	RA
GRArg01	-	-	Greece	Argolis, Mt Artemisio	37.65254	22.85822	2008	0.34	GH
GRCr01*	-	-	Greece	Crete	35.24012	24.80927	1934	0.26	GH
**OD067***	Diakopto	-	Greece	Diakopto	39.76444	19.58944	1938	7.63	GB
**OD070***	Diakopto	-	Greece	Diakopto	39.76444	19.58944	1938	11.76	GB
**OD064b***	Chelmos	-	Greece	Mt. Chelmos, nr. Kalavryta	38.03328	22.11035	1938	13.67	GB
**OD076***	-	-	Greece	Mt. Chelmos, nr. Kalavryta	38.03328	22.11035	1938	5.40	GB
**OD068***	-	-	Greece	Mt. Hortiat, nr. Salonika	40.64006	22.94442	1938	0.28	GB
**OD074***	-	-	Greece	Mt. Hortiat, nr. Salonika	40.64006	22.94442	1938	1.27	GB
GRNk01	-	-	Greece	Nea Karvali	40.96242	24.50827	2008	1.32	GH
**GRTz01**	-	-	Greece	Tzia Island, Vorukari-Otzias	37.61667	24.33333	2005	9.23	GH
**HFü01***	-	-	Hungary	Fülöphaza	46.89140	19.44325	1977	0.26	GH
HFü02*	-	-	Hungary	Fülöphaza	46.89140	19.44325	1977	0.53	GH
**ICo10**	Cogne	-	Italy	Basse Valle di Cogne	45.60762	7.35909	2005	9.75	GH
**ICo20**	Cogne	-	Italy	Basse Valle di Cogne	45.60762	7.35909	2005	26.10	GH
**IPo01***	Popoli	-	Italy	Capo Pescara, Popoli	42.20057	13.88168	1992	1.32	GH

IGr01*	-	-	Italy	Caulonia, Monte Gremi	38.38068	16.40959	1948	13.71	GH
**IGr02***	Caulonia	-	Italy	Caulonia, Monte Gremi	38.38068	16.40959	1948	33.74	GH
ICe01*	-	-	Italy	Cetraro, Calabria	39.51655	15.94091	1948	0.15	GH
**ICe02***	Certaro	-	Italy	Cetraro, Calabria	39.51655	15.94091	1948	12.13	GH
ISN03*	-	-	Italy	Isola Tremiti, San Nicola	42.12042	15.50385	1954	0.26	GH
**ISN04***	Tremiti	-	Italy	Isola Tremiti, San Nicola	42.12042	15.50385	1954	36.90	GH
**ICp03***	Lampedusa	-	Italy	Lampedusa, Capo Peneto	35.50862	12.59292	1969	20.82	GH
**IRa01***	Randazzo	-	Italy	Randazzo, Etna	37.75101	14.99344	1969	46.66	GH
**IRa02***	Randazzo	-	Italy	Randazzo, Etna	37.75101	14.99344	1969	55.62	GH
**IRN01***	Sicily	-	Italy	Rocca di Novara, Sicily	37.99572	15.14656	1967	64.58	GH
**IRN02***	Sicily	-	Italy	Rocca di Novara, Sicily	37.99572	15.14656	1967	5.80	GH
**ISu01**	Susa	-	Italy	Susa	45.13861	7.04846	2009	63.26	GH
**ISu03**	Susa	-	Italy	Susa	45.13861	7.04846	2009	52.46	GH
**IMa03**	Udine	-	Italy	Udine, Magredi di Cordenons	45.98584	12.70355	2009	69.85	GH
**IMa10**	Udine	-	Italy	Udine, Magredi di Cordenons	45.98584	12.70355	2009	87.52	GH
IVi01*	-	-	Italy	Veneto, Verona, Villafranca	45.35526	10.84743	1946	1.05	GH
IVi02*	-	-	Italy	Veneto, Verona, Villafranca	45.35526	10.84743	1946	8.17	GH
**MDas01***	Dashinchilen	-	Mongolia	Dashinchilen	47.85074	104.04213	1968	34.80	GH
**MDas02***	Dashinchilen	-	Mongolia	Dashinchilen	47.85074	104.04213	1968	50.87	GH
**MZo01***	Zogt-Ovoo	-	Mongolia	Zogt-Ovoo	44.42490	105.32154	1967	54.30	GH
**MZo02***	Zogt-Ovoo	-	Mongolia	Zogt-Ovoo	44.42490	105.32154	1967	28.73	GH
**OD079***	-	-	Morocco	10 km S. of Fez	34.01813	-5.00785	1968	0.64	GB
**OD122***	-	-	Morocco	10 km S. of Fez	34.01813	-5.00785	1968	0.95	GB
OD077*	-	-	Morocco	Ijoukak	30.99732	-8.16254	1936	1.27	GB
OD113*	-	-	Morocco	Ijoukak	30.99732	-8.16254	1936	0.95	GB
OD005*	-	-	Perse	Rudhend-Delidjai	35.68920	51.38897	1948	0.83	DB
OD006*	-	-	Perse	Rudhend-Delidjai	35.68920	51.38897	1948	0.00	DB
OD069*	Boca	-	Portugal	Boca dos Corgos, Madeira	32.76071	-16.95947	1978	12.08	GB
OD071*	Boca	-	Portugal	Boca dos Corgos, Madeira	32.76071	-16.95947	1978	27.34	GB
OD158*	Encumenda	-	Portugal	Encumenda, Madeira	32.76071	-16.95947	1954	0.32	GB
OD157*	Mangaulde	-	Portugal	Mangaulde	40.60837	-7.76642	1969	25.11	GB
OD066*	Paul	-	Portugal	Paul da Serra, Madeira	32.73333	17.05000	1935	1.27	GB
OD084*	Paul	-	Portugal	Paul da Serra, Madeira	32.73333	17.05000	1964	9.54	GB
OD082*	Areiro	-	Portugal	Pico de Areiro, Madeira	32.71216	-16.88835	1980	121.44	GB
OD088*	Areiro	-	Portugal	Pico de Areiro, Madeira	32.71216	-16.88835	1980	58.81	GB
OD090*	Areiro	-	Portugal	Pico de Areiro, Madeira	32.71216	-16.88835	1980	31.47	GB
OD142*	-	-	Portugal	Serra da Estrella	40.32187	-7.61297	1933	16.21	GB
OD146*	Estrella	-	Portugal	Serra da Estrella	40.32187	-7.61297	1933	14.94	GB
**OD153***	Estrella	-	Portugal	Tapa da Ajuda	38.71131	-9.19821	1934	9.85	GB
**RKur04**	Kurgan	-	Russia	Kurgan	55.46491	65.30535	2009	49.03	GH
RKur05	-	-	Russia	Kurgan	55.46491	65.30535	2009	58.26	GH
SKSo02*	-	-	Slovakia	Somotor	48.39932	21.80894	1951	1.05	GH
**EPu01***	Granada	-	Spain	Granada, Puerto de la Mora	37.17734	-3.59856	1965	3.16	GH
EPu02*	-	-	Spain	Granada, Puerto de la Mora	37.17734	-3.59856	1965	1.32	GH
**OD046***	Guadarrama	-	Spain	Sierra de Guadarrama	40.85090	-3.94997	1958	4.77	RE
**OD063***	Guadarrama	-	Spain	Sierra de Guadarrama	40.85090	-3.94997	1958	5.72	RE
**ECa06**	Capilieira	-	Spain	Sierra Nevada, Capileira	37.05000	-3.30000	2007	15.29	RA
**ECa09**	Capilieira	-	Spain	Sierra Nevada, Capileira	37.05000	-3.30000	2007	105.70	RA

OD073*	-	-	Spain	Tenerife, Canary Is.	28.45151	-16.36750	1966	10.49	GB
**OD089***	Canary	-	Spain	Tenerife, Canary Is.	28.45151	-16.36750	1966	2.54	GB
**006***	Ausserberg	Ausserberg	Switzerland	Ausserberg	46.31444	7.85070	1963	12.18	HB
013*	-	-	Switzerland	Branson	46.12998	7.09250	1931	0.62	HB
014*	-	-	Switzerland	Branson	46.12998	7.09250	1931	0.83	HB
**007***	Finges	Finges 1954	Switzerland	Finges	46.29393	7.55920	1954	22.70	HB
**3609***	Finges	Finges 1950	Switzerland	Finges	46.29393	7.55920	1949	26.83	AF
**3610***	Finges	Finges 1950	Switzerland	Finges	46.29393	7.55920	1949	7.84	AF
3611*	-	-	Switzerland	Finges	46.29393	7.55920	1949	14.65	AF
**3612***	Finges	Finges 1950	Switzerland	Finges	46.29393	7.55920	1949	12.18	AF
**3613***	Finges	Finges 1950	Switzerland	Finges	46.29393	7.55920	1949	9.29	AF
3614*	-	-	Switzerland	Finges	46.29393	7.55920	1949	14.86	AF
**3615***	Finges	Finges 1950	Switzerland	Finges	46.29393	7.55920	1949	19.61	AF
3616*	-	-	Switzerland	Finges	46.29393	7.55920	1949	0.28	AF
3618*	-	-	Switzerland	Finges	46.29393	7.55920	1949	0.29	AF
3619*	-	-	Switzerland	Finges	46.29393	7.55920	1949	0.39	AF
3621*	-	-	Switzerland	Finges	46.29393	7.55920	1949	0.00	AF
**3623***	Finges	Finges 1950	Switzerland	Finges	46.29393	7.55920	1949	42.11	AF
**3624***	Finges	Finges 1950	Switzerland	Finges	46.29393	7.55920	1949	36.53	AF
OD025*	-	-	Switzerland	Finges	46.29393	7.55920	1939	0.95	PS
OD026*	-	-	Switzerland	Finges	46.29393	7.55920	1939	1.27	PS
OD027*	-	-	Switzerland	Finges	46.29393	7.55920	1939	0.64	PS
**OD028***	Finges	Finges 1940	Switzerland	Finges	46.29393	7.55920	1939	2.23	PS
OD029*	-	-	Switzerland	Finges	46.29393	7.55920	1939	1.46	PS
OD030*	-	-	Switzerland	Finges	46.29393	7.55920	1942	0.32	PS
OD032*	-	-	Switzerland	Finges	46.29393	7.55920	1939	2.23	PS
OD033*	-	-	Switzerland	Finges	46.29393	7.55920	1939	0.64	PS
OD034*	-	-	Switzerland	Finges	46.29393	7.55920	1939	1.59	PS
OD035*	Finges	Finges 1940	Switzerland	Finges	46.29393	7.55920	1939	4.13	PS
OD036*	Finges	Finges 1940	Switzerland	Finges	46.29393	7.55920	1939	4.13	PS
OD038*	-	-	Switzerland	Finges	46.29393	7.55920	1939	1.27	PS
OD042*	-	-	Switzerland	Finges	46.29393	7.55920	1939	1.91	PS
OD043*	-	-	Switzerland	Finges	46.29393	7.55920	1939	3.81	PS
OD044*	Finges	Finges 1940	Switzerland	Finges	46.29393	7.55920	1939	2.23	PS
OD072*	-	-	Switzerland	Finges	46.29393	7.55920	1939	3.81	GB
OD163*	Finges	Finges 1940	Switzerland	Finges	46.29393	7.55920	1939	1.27	GB
012*	Follatères	Follatères	Switzerland	Follatères	46.10853	7.07252	1931	0.83	HB
1	Gampel	Gampel 2005	Switzerland	Gampel	46.31578	7.74194	2005	36.20	HB
CHGa01	Gampel	Gampel 2005	Switzerland	Gampel	46.31578	7.74194	2005	7.12	RA
CHGa02	Gampel	Gampel 2005	Switzerland	Gampel	46.31578	7.74194	2005	10.54	RA
CHGa03	Gampel	Gampel 2005	Switzerland	Gampel	46.31578	7.74194	2005	10.28	RA
CHGa04	-	-	Switzerland	Gampel	46.31578	7.74194	2005	8.96	RA
CHGa05	Gampel	Gampel 2005	Switzerland	Gampel	46.31578	7.74194	2005	12.92	RA
CHGa06	Gampel	Gampel 2005	Switzerland	Gampel	46.31578	7.74194	2005	17.66	RA
CHGa07	Gampel	Gampel 2005	Switzerland	Gampel	46.31578	7.74194	2005	15.82	RA
CHGa08	Gampel	Gampel 2005	Switzerland	Gampel	46.31578	7.74194	2005	13.97	RA
CHGa09	Gampel	Gampel 2005	Switzerland	Gampel	46.31578	7.74194	2005	3.69	RA
CHGa10	Gampel	Gampel 2005	Switzerland	Gampel	46.31578	7.74194	2005	3.16	RA

CHGa11	Gampel	Gampel 2005	Switzerland	Gampel	46.31578	7.74194	2005	7.38	RA
CHGa12	-	-	Switzerland	Gampel	46.31578	7.74194	2005	46.92	RA
CHGa13	Gampel	Gampel 2005	Switzerland	Gampel	46.31578	7.74194	2005	36.64	RA
CHGa14	-	-	Switzerland	Gampel	46.31578	7.74194	2005	7.38	RA
CHGa15	Gampel	Gampel 2005	Switzerland	Gampel	46.31578	7.74194	2005	63.53	RA
CHGa16	Gampel	Gampel 2005	Switzerland	Gampel	46.31578	7.74194	2005	10.28	RA
CHGa17	Gampel	Gampel 2005	Switzerland	Gampel	46.31578	7.74194	2005	9.38	RA
CHGa18	Gampel	Gampel 2005	Switzerland	Gampel	46.31578	7.74194	2005	33.21	RA
CHGa19	Gampel	Gampel 2005	Switzerland	Gampel	46.31578	7.74194	2005	12.92	RA
CHGa20	Gampel	Gampel 2005	Switzerland	Gampel	46.31578	7.74194	2005	14.23	RA
OD050	-	-	Switzerland	Lugano	46.00368	8.95105	-	1.91	RE
3626*	-	-	Switzerland	Martigny	46.10498	7.07553	1938	0.83	AF
OD018*	-	-	Switzerland	Ob-Waiden	46.31285	7.97237	1905	1.91	PS
OD024*	-	-	Switzerland	Ob-Waiden	46.31285	7.97237	1905	0.64	PS
3617*	Saas	Saas	Switzerland	Saas	46.10911	7.92971	1938	5.57	AF
017*	-	-	Switzerland	Sierre	46.29413	7.53354	1884	3.10	HB
OD010*	Sierre	Sierre 1940	Switzerland	Sierre	46.29413	7.53354	1941	3.30	PS
OD011*	-	-	Switzerland	Sierre	46.29413	7.53354	1908	0.83	PS
OD013*	-	-	Switzerland	Sierre	46.29413	7.53354	1908	0.62	PS
OD015*	-	-	Switzerland	Sierre	46.29413	7.53354	1908	0.41	PS
OD016*	-	-	Switzerland	Sierre	46.29413	7.53354	1908	0.83	PS
OD019*	-	-	Switzerland	Sierre	46.29413	7.53354	1908	0.64	PS
OD020*	-	-	Switzerland	Sierre	46.29413	7.53354	1908	0.64	PS
OD022*	-	-	Switzerland	Sierre	46.29413	7.53354	1941	0.64	PS
OD023*	Sierre	Sierre 1940	Switzerland	Sierre	46.29413	7.53354	1941	8.90	PS
OD031*	-	-	Switzerland	Sierre	46.29413	7.53354	1941	6.99	PS
OD037*	Sierre	Sierre 1940	Switzerland	Sierre	46.29413	7.53354	1941	3.81	PS
OD039*	-	-	Switzerland	Sierre	46.29413	7.53354	1941	15.90	PS
OD040*	Sierre	Sierre 1940	Switzerland	Sierre	46.29413	7.53354	1941	1.59	PS
OD041*	-	-	Switzerland	Sierre	46.29413	7.53354	1941	0.64	PS
OD045*	-	-	Switzerland	Sierre	46.29413	7.53354	1908	0.32	BO
OD047*	Sierre	Sierre 1908	Switzerland	Sierre	46.29413	7.53354	1908	1.91	BO
OD048*	-	-	Switzerland	Sierre	46.29413	7.53354	1908	0.95	RE
OD049*	-	-	Switzerland	Sierre	46.29413	7.53354	1908	1.27	BO
OD051	-	-	Switzerland	Sierre	46.29413	7.53354	-	1.91	RE
OD052*	-	-	Switzerland	Sierre	46.29413	7.53354	1908	0.95	BO
OD053*	-	-	Switzerland	Sierre	46.29413	7.53354	1908	0.95	BO
OD056*	-	-	Switzerland	Sierre	46.29413	7.53354	1908	1.27	BO
OD059*	-	-	Switzerland	Sierre	46.29413	7.53354	1908	0.95	RE
OD061*	-	-	Switzerland	Sierre	46.29413	7.53354	1908	0.32	RE
OD062*	-	-	Switzerland	Sierre	46.29413	7.53354	1908	1.27	BO
OD064a*	-	-	Switzerland	Sierre	46.29413	7.53354	1908	0.64	BO
CHCo01	-	-	Switzerland	St-Martin, Combioula	46.17247	7.42145	2005	20.98	RA
CHCo02	-	-	Switzerland	St-Martin, Combioula	46.17247	7.42145	2005	0.32	RA
CHCo03	Lower Hérens V.	Lower Hérens V. 2005	Switzerland	St-Martin, Combioula	46.17247	7.42145	2005	3.50	RA
CHCo04	Lower Hérens V.	Lower Hérens V. 2005	Switzerland	St-Martin, Combioula	46.17247	7.42145	2005	19.71	RA
CHCo05	Lower Hérens V.	Lower Hérens V. 2005	Switzerland	St-Martin, Combioula	46.17247	7.42145	2005	10.17	RA
CHCo06	Lower Hérens V.	Lower Hérens V. 2005	Switzerland	St-Martin, Combioula	46.17247	7.42145	2005	20.35	RA

CHCo07	Lower Hérens V.	Lower Hérens V. 2005	Switzerland	St-Martin, Combioula	46.17247	7.42145	2005	6.26	RA
CHCo08	Lower Hérens V.	Lower Hérens V. 2005	Switzerland	St-Martin, Combioula	46.17247	7.42145	2005	18.44	RA
CHOs01	Lower Hérens V.	Lower Hérens V. 2005	Switzerland	St-Martin, Ossona	46.18291	7.42343	2005	13.18	RA
CHOs02	-	-	Switzerland	St-Martin, Ossona	46.18291	7.42343	2005	11.60	RA
CHOs03	Lower Hérens V.	Lower Hérens V. 2005	Switzerland	St-Martin, Ossona	46.18291	7.42343	2005	71.44	RA
CHOs04	-	-	Switzerland	St-Martin, Ossona	46.18291	7.42343	2005	10.54	RA
CHOs05	Lower Hérens V.	Lower Hérens V. 2005	Switzerland	St-Martin, Ossona	46.18291	7.42343	2005	26.80	RA
CHOs06	Lower Hérens V.	Lower Hérens V. 2005	Switzerland	St-Martin, Ossona	46.18291	7.42343	2005	14.00	RA
CHOs07	Lower Hérens V.	Lower Hérens V. 2005	Switzerland	St-Martin, Ossona	46.18291	7.42343	2005	21.09	RA
CHOs08	Lower Hérens V.	Lower Hérens V. 2005	Switzerland	St-Martin, Ossona	46.18291	7.42343	2005	6.06	RA
CHOs09	Lower Hérens V.	Lower Hérens V. 2005	Switzerland	St-Martin, Ossona	46.18291	7.42343	2005	9.49	RA
CHOs10	Lower Hérens V.	Lower Hérens V. 2005	Switzerland	St-Martin, Ossona	46.18291	7.42343	2005	8.00	RA
CHOs11	Lower Hérens V.	Lower Hérens V. 2005	Switzerland	St-Martin, Ossona	46.18291	7.42343	2005	14.76	RA
CHOs13	Lower Hérens V.	Lower Hérens V. 2005	Switzerland	St-Martin, Ossona	46.18291	7.42343	2005	15.29	RA
016*	St-Niklaus	St-Niklaus	Switzerland	St-Niklaus	46.17623	7.80459	1927	2.89	HB
015*	-	-	Switzerland	Stalden	46.23310	7.87067	1886	6.60	HB
OD095*	-	-	Turkey	Elmadag	39.02020	31.15100	1969	0.44	GB
OD105*	-	-	Turkey	Elmadag	39.02020	31.15100	1969	0.32	GB
OD138*	-	-	Turkey	Kars, Igdir	39.92006	44.04362	1960	1.91	GB
OD147*	Malatya	-	Turkey	Malatya	38.35536	38.33353	1930	16.85	GB
OD065*	-	-	Turkey	Mugla Vilayet	37.18358	28.48640	1947	1.91	GB
OD115*	Mugla	-	Turkey	Mugla Vilayet	37.18358	28.48640	1947	44.51	GB
OD133*	Niksar	-	Turkey	Niksar	40.59016	36.95119	1959	10.17	GB
TSV01*	Sivrihisar	-	Turkey	Sivrihisar	39.45130	31.53780	1969	50.35	GH
TSV02*	-	-	Turkey	Sivrihisar	39.45130	31.53780	1969	1.85	GH
OD075*	-	-	Turkey	Urfa	37.16740	38.79552	1931	7.31	GB
OD087*	Urfa	-	Turkey	Urfa	37.16740	38.79552	1931	2.54	GB
OD091*	-	-	Turkey	Yakacik	41.00824	28.97836	1959	0.95	GB

Table S2 Samples used for the generation of the probes in the hyRAD protocol. Given are sample label, sampling site (country, location, and coordinates) and collection year. All samples were provided by Gerald Heckel's lab (University of Bern, Switzerland).

Label	Country	Location	Latitude	Longitude	Year
RKuR4	Russia	Stawropol	55.4649	65.3054	2009
CHOs12	Switzerland	Ossona	46.1829	7.4234	2005
HRVo02	Croatia	Voz	45.2319	14.5796	2005
ECa02	Spain	Capileira	37.0500	-3.3000	2007

Table S3 Scenarios of population decline tested for the Finges population between 1940 and 1950 as well as the associated statistic for the comparison between the simulated and observed minor allele frequency distribution. N_i and N_f are respectively the initial and the final population sizes, decline is the type of decline simulated and the D statistic is the corresponding Kolmogorov-Smirnov statistic. All observed and simulated distributions were significantly different ($\alpha = 0.05$) after standard false discovery rate correction (Benjamini & Hochberg 1995).

N_i	N_f	Decline	D statistic
100	10	Linear	0.53*
100	10	Exponential weak	0.52*
100	10	Exponential strong	0.52*
500	10	Linear	0.51*
500	10	Exponential weak	0.51*
500	10	Exponential strong	0.50*
1000	10	Linear	0.48*
1000	10	Exponential weak	0.50*
1000	10	Exponential strong	0.52*
10000	10	Linear	0.46*
10000	10	Exponential weak	0.50*
10000	10	Exponential strong	0.50*
500	100	Linear	0.46*
500	100	Exponential weak	0.46*
500	100	Exponential strong	0.46*
1000	100	Linear	0.46*
1000	100	Exponential weak	0.46*
1000	100	Exponential strong	0.46*
10000	100	Linear	0.46*
10000	100	Exponential weak	0.46*
10000	100	Exponential strong	0.46*
1000	500	Linear	0.46*
1000	500	Exponential weak	0.46*
1000	500	Exponential strong	0.46*
10000	500	Linear	0.46*
10000	500	Exponential weak	0.46*
10000	500	Exponential strong	0.46*
10000	1000	Linear	0.46*
10000	1000	Exponential weak	0.46*
10000	1000	Exponential strong	0.46*

Table S4 Number of polymorphic sites after each filtering step for both spatial and temporal dataset. Initial is the number of polymorphic sites before filtering, QUAL > 30 is after removing sites with a quality below 30; indels is after removing indels; bi-allelic is after removing sites with more than two alleles; MAC > 6 is after removing sites with a minor allele count lower than 6; max-missing < 50% is after removing sites present in less than 50% of the samples; depth > 6 is after removing sites with a depth value below 6; QUAL/DP > 0.25 is after removing sites with a quality/depth ratio inferior to 0.25 and paralogs corresponds to a removal of paralogous sites.

	Spatial analysis	Temporal analysis
Initial	1 730 734	278 634
QUAL > 30	112 415	73 438
Indels	53 537	37 222
Bi-allelic	53 212	37 088
MAC > 6	41 385	25 996
Max-missing < 50%	26 548	17 475
Depth > 6	3 850	2 366
QUAL/DP > 0.25	1 490	1 816
Paralogs	1 165	1 444

SUPPLEMENTARY FIGURES

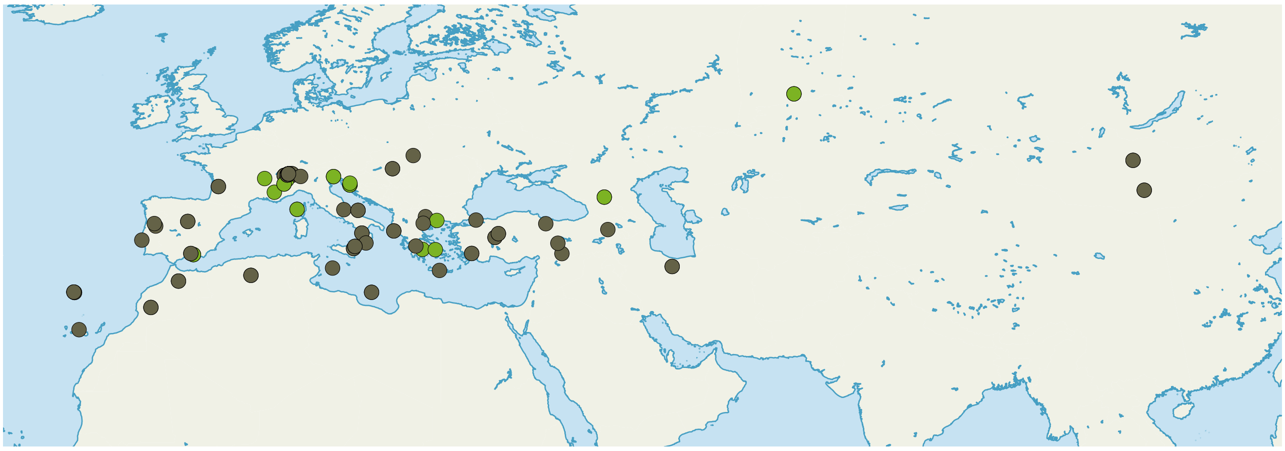

Figure S1 Spatial distribution of all available *O.decorus* samples across its distribution range in Europe, northern Africa and central Asia. Green represents fresh samples and brown historical samples.

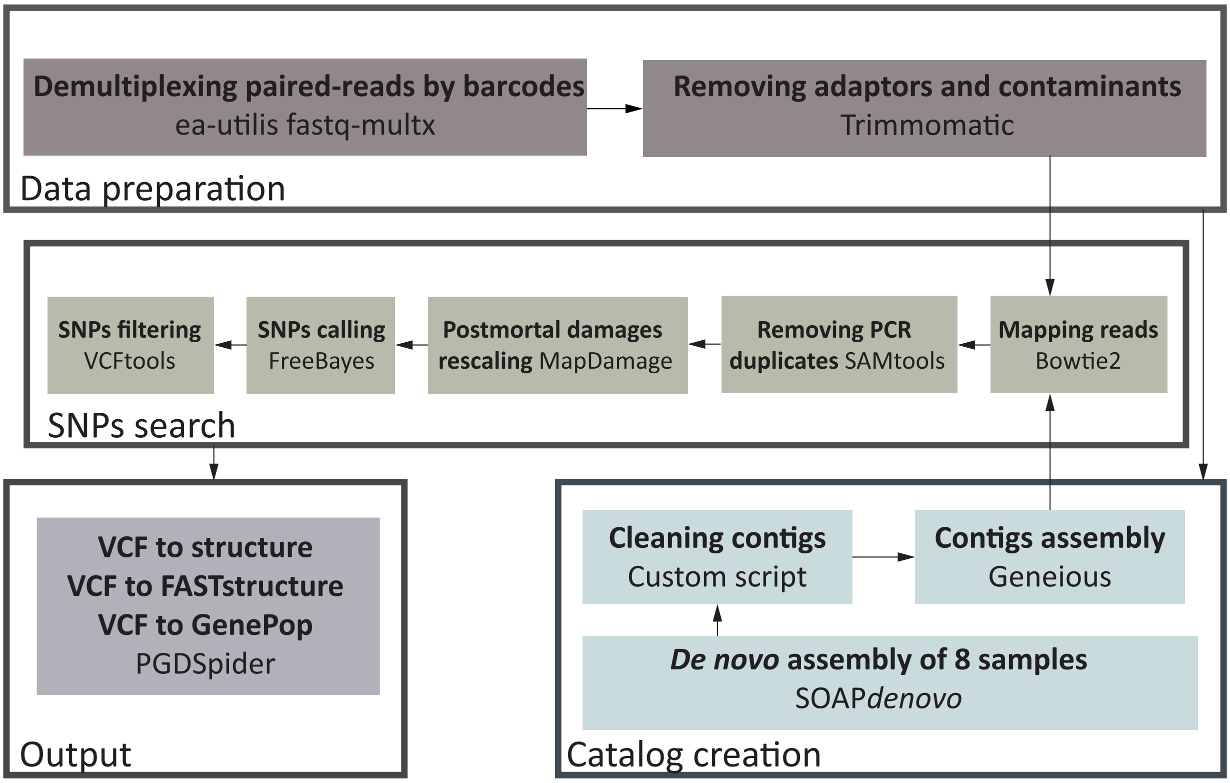

Figure S2 Custom bioinformatics pipeline to retrieve SNPs from hyRAD data. For the temporal analysis of Swiss samples, adaptors and contaminants removal, reads mapping, PCR duplicates removal and postmortal damages rescaling were performed with the PALEOMIX BAM pipeline (Schubert *et al.* 2014).

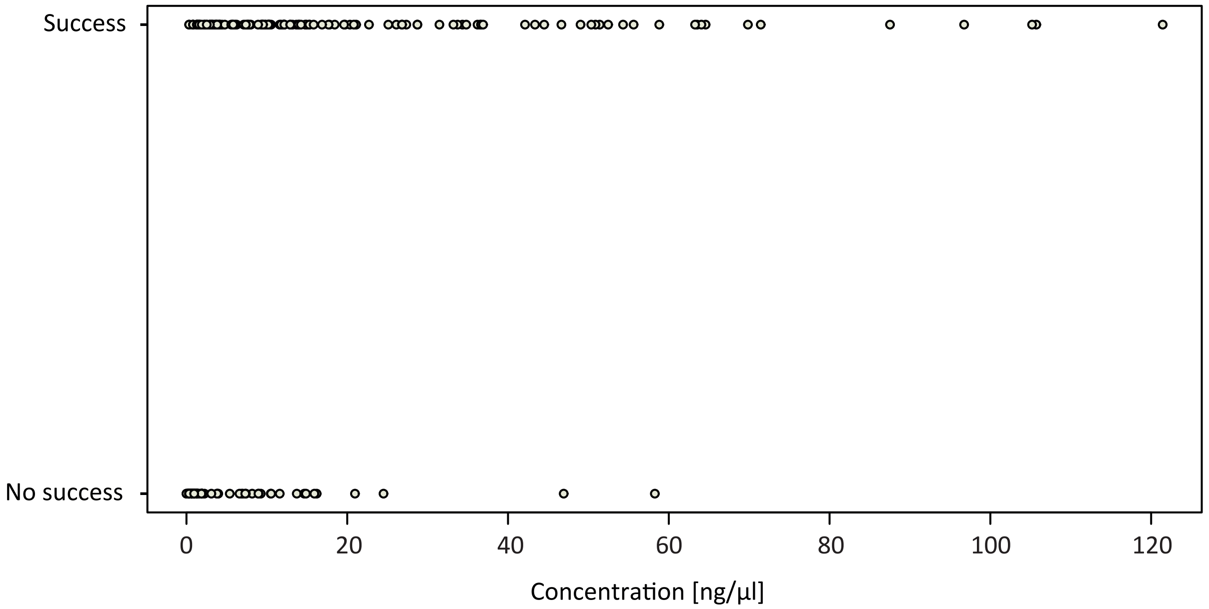

Figure S3 Success of sequencing according to the DNA concentration of each sample. Concentration has a significant effect on success of sequencing ($\chi^2 = 33.4$, df = 1, p = 8e-9).

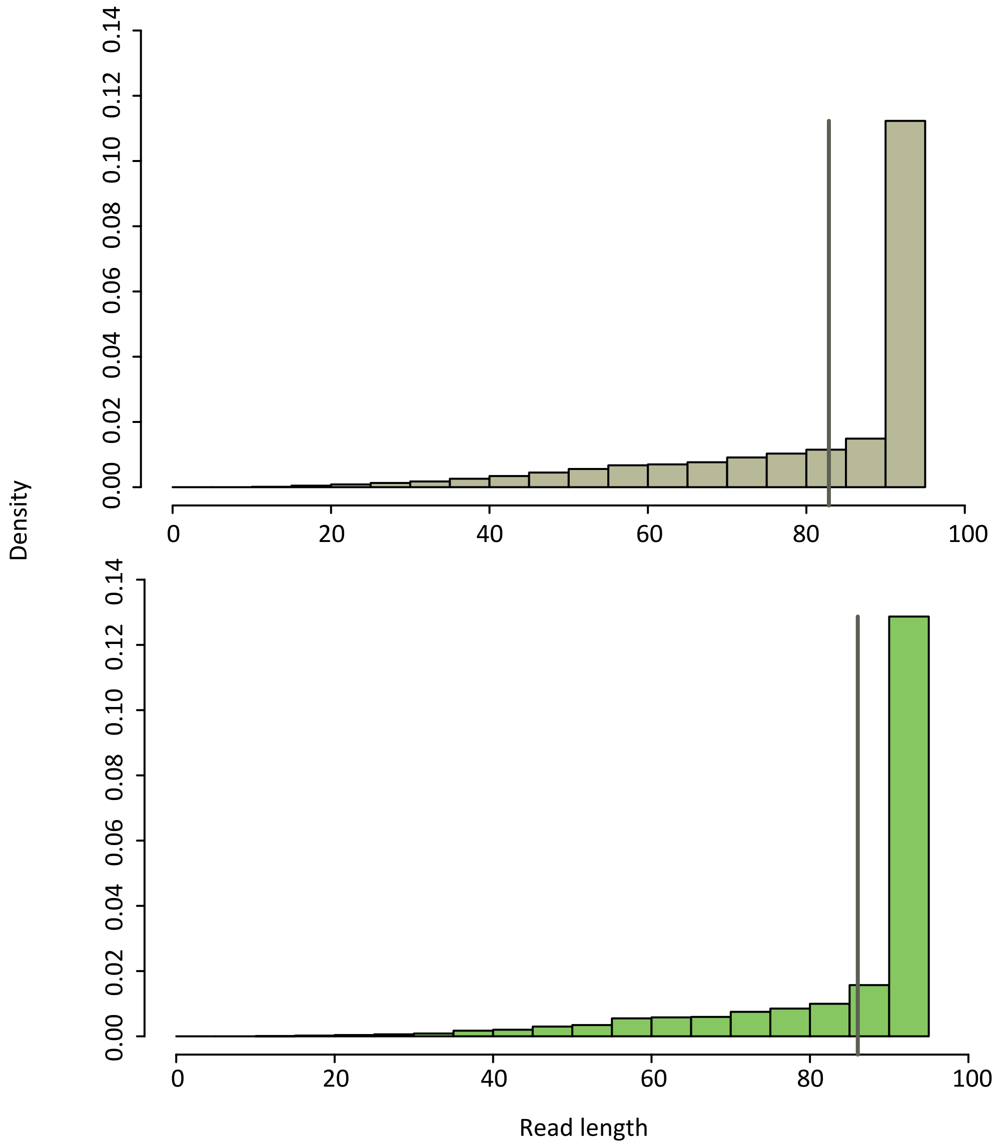

Figure S4 Distribution of read length for all sequenced historical (n = 68; brown) and contemporary (n = 51; green) samples. The vertical line indicates the mean read length. There was no significant difference between the two groups (W = 4190.5, p = 0.14).

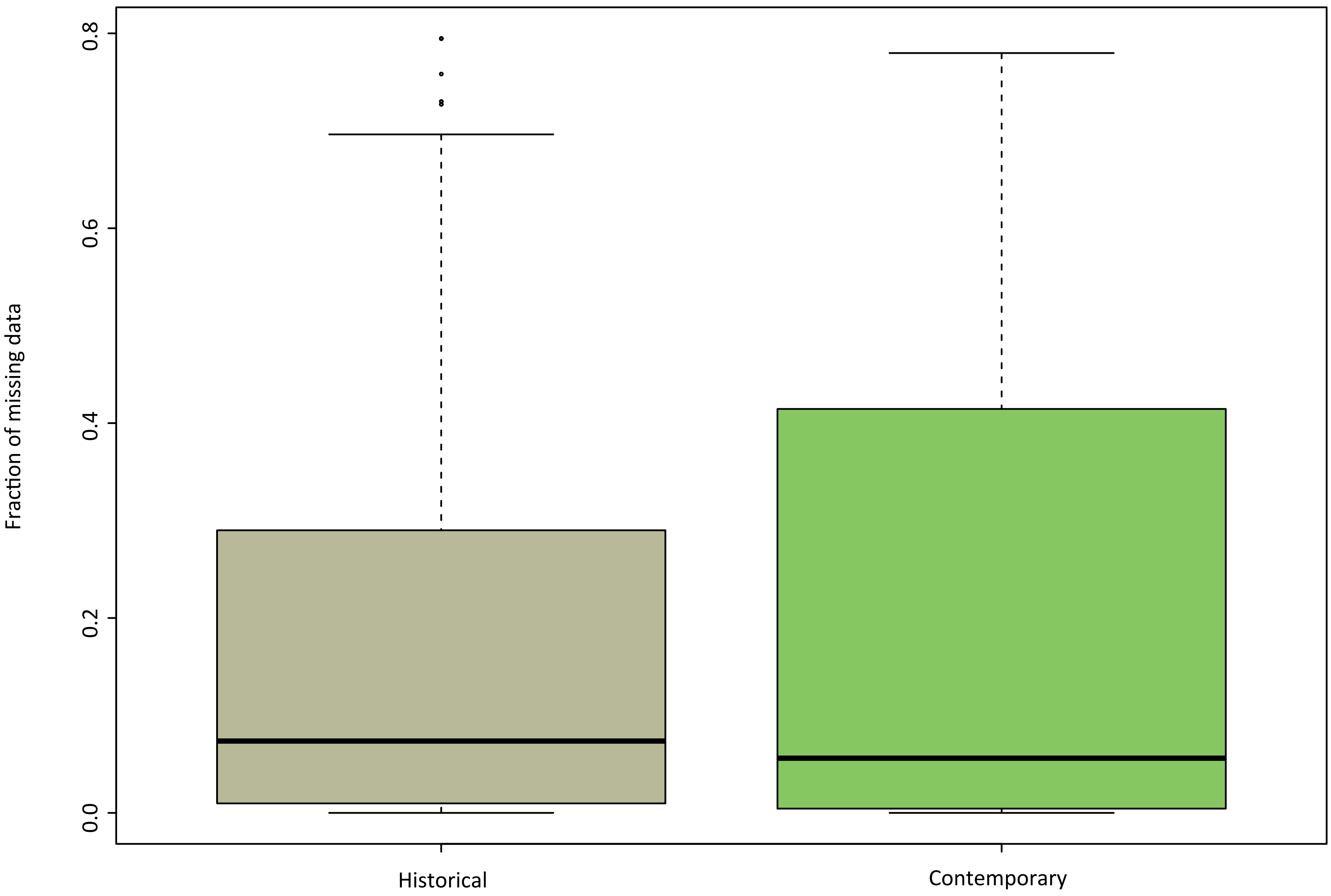

Figure S5 Distribution of the fraction of missing data for all sequenced historical (n = 68; brown) and contemporary (n = 51; green) samples. Top and bottom of the box respectively represent the upper and the lower quartile and the bold line corresponds to the median. No significant difference was found between the two groups (W = 1818.5, p = 0.72).

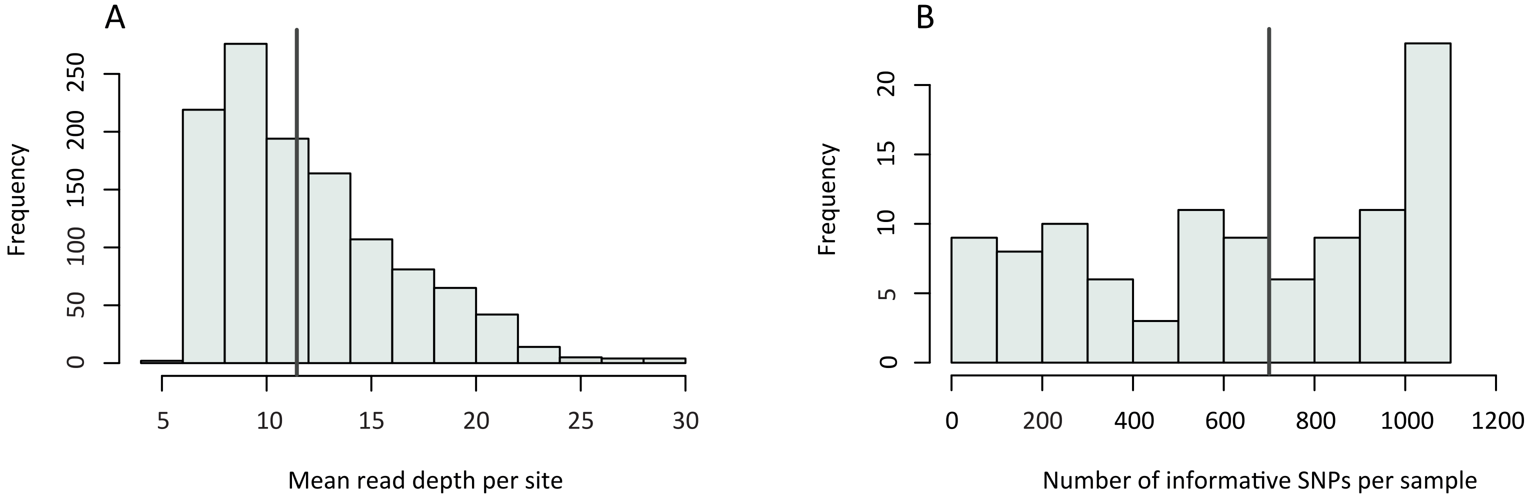

Figure S6 (A) Distribution of mean read depth per site and (B) informative SNPs per sample for the 119 samples originating throughout the species geographical range. The vertical line represents the mean.

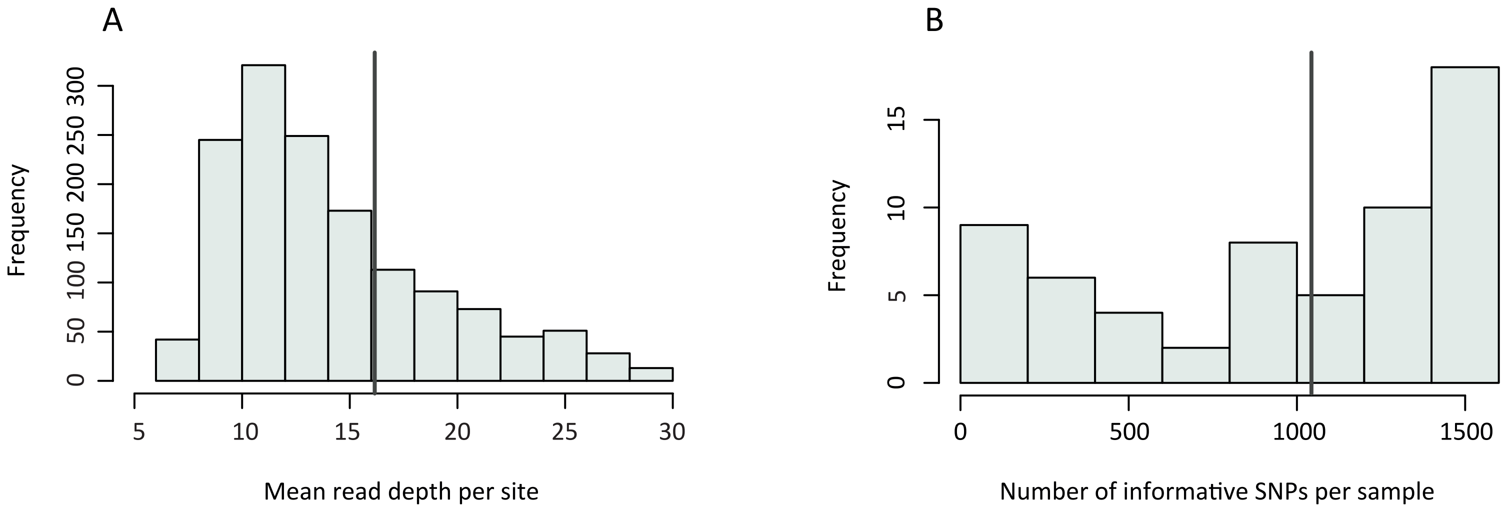

Figure S7 (A) Distribution of mean read depth per site and (B) informative SNPs per sample for the 62 Swiss samples. The vertical line represents the mean.

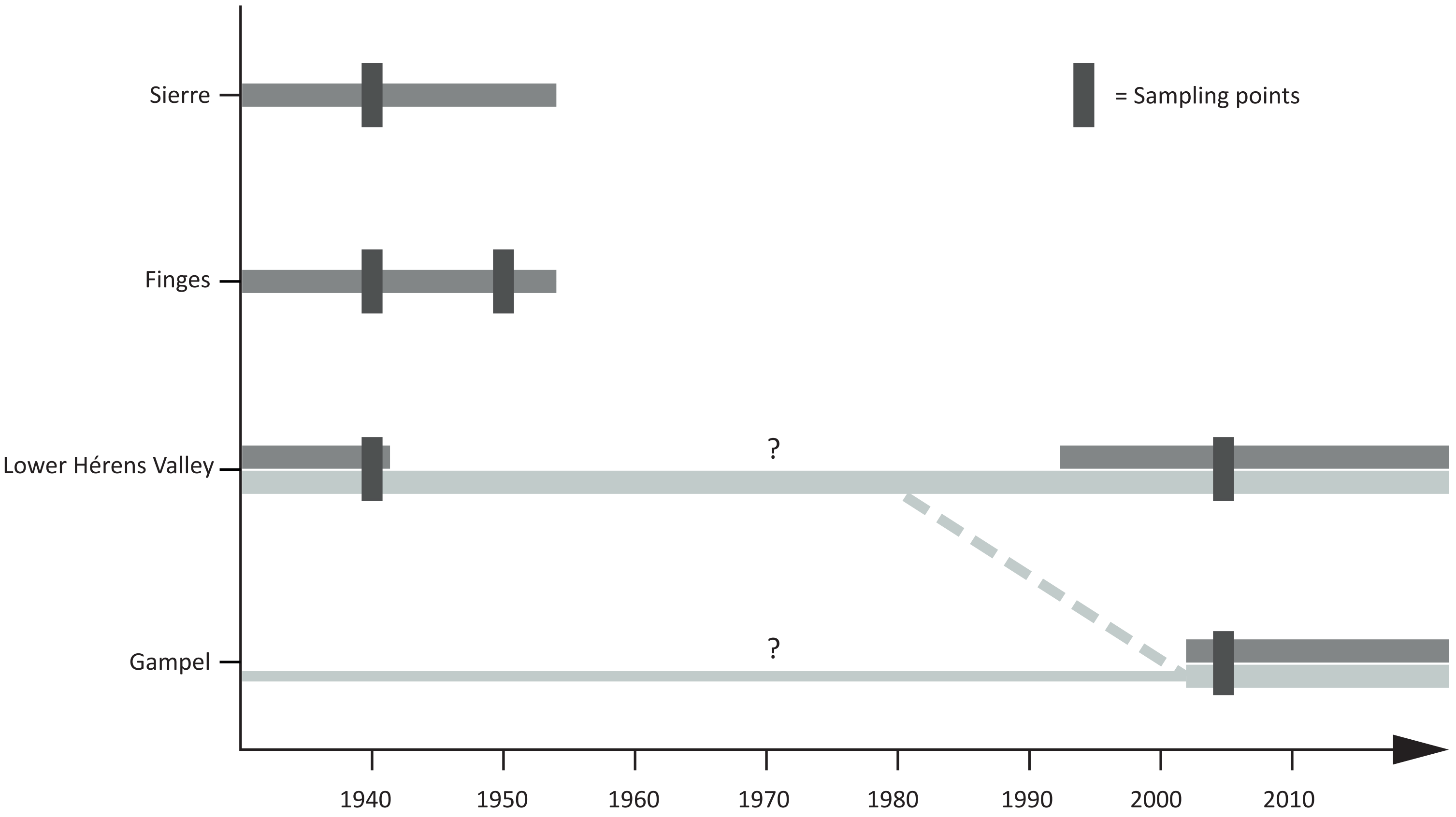

Figure S8 Timeframe illustrating the Swiss *O. decorus* populations used in this study. Dark grey lines represent the known presence of the species in Switzerland according to the Swiss Center for Fauna Cartography (CSCF). Possible presence of the species in Switzerland according to genetic data is indicated in light grey. Question marks indicate that the species was considered as absent between 1940-1992. Dashed lines stand for intentional transfer or natural dispersal of individuals.

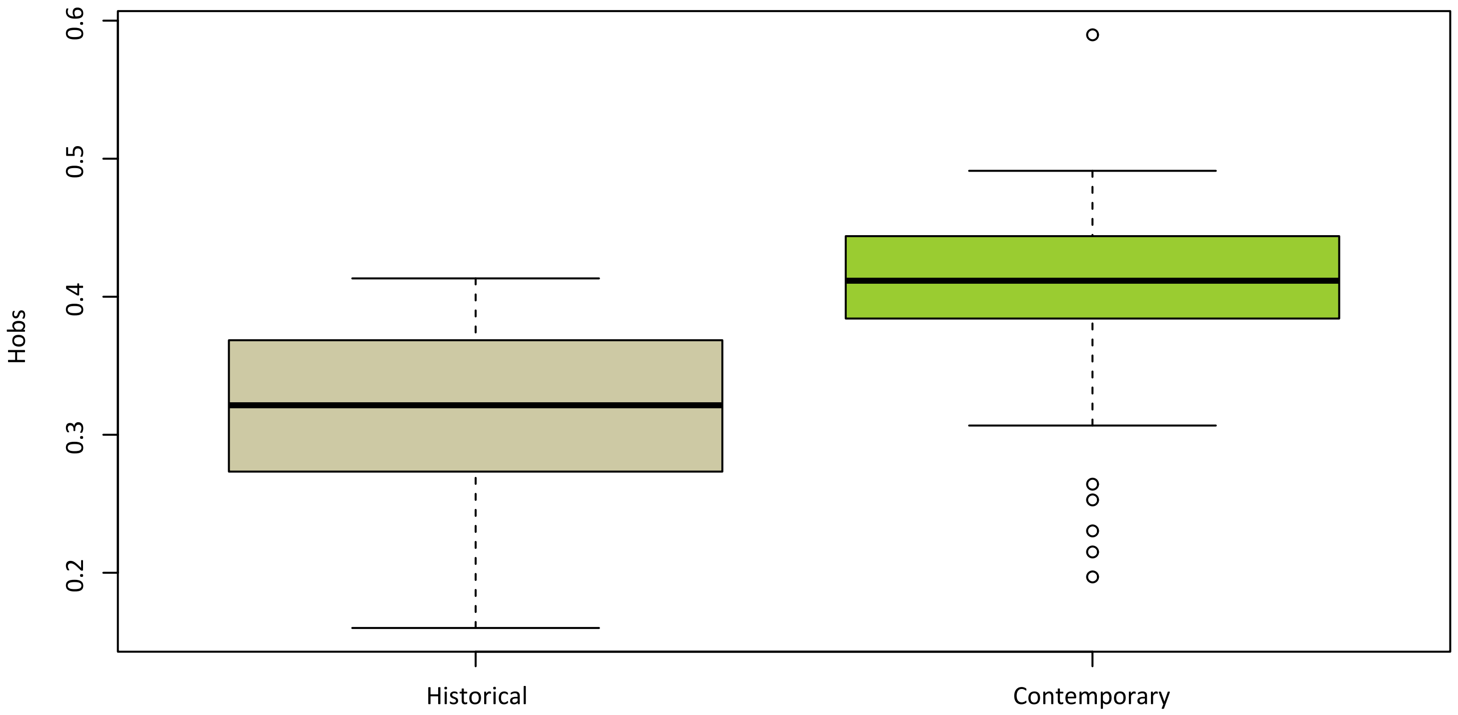

Figure S9 Distribution of observed heterozygosities at the individual level both in historical (n = 29) and contemporary (n = 33) samples when PCR duplicates are not removed carefully. Top and bottom of the box respectively represent the upper and the lower quartile and the bold line corresponds to the median.

APPENDICES

Appendix S1 Blast search against GenBank and matching sequences extraction.

This Perl script takes as input a set of contigs, blasts them against GenBank and extracts sequences of interest using a keyword against taxonomic identifications.

```
## usage: perl QueryAnnot.pl data.in/Contigs.fasta Pinales keepall|hits

my $file = $ARGV[0]; #contig file, must be in data.in/, formatted as from Geneious assemblies.

my $keyword = $ARGV[1]; #keyword to look after, and extract contigs that match with it.

my $mode = $ARGV[2]; #either CompleteSeq = extract complete sequence or HitsOnly = keep only region generating a blast hit

chomp($mode);


## Important params (hard coded here)

# blast searches

my $nthreads = 4; #nthreads for blast search

my $eval = 0.1; #min e-value for blast search

my $pctsim = 30; #min % simil for blast search


#### checks

if($mode ne "CompleteSeq" && $mode ne "HitsOnly"){

  print "STOP\: Incorrect analysis mode, make sure to use either CompleteSeq or HitsOnly mode!\n";

  exit;

  }


print "\n###### QueryAnnot Pipeline ######\nContigs file: $file\nBlast params:\n-threads: $nthreads\n-evalue: $eval\n-pctsimil: $pctsim\n\nAnalysis mode: $mode\n\n\n\n";


#### Settings

use File::Basename;

# paths to databases

my $db = "/data/GenBank/db/nt";

my $refpath = "/archive/Scripts_NGS/AmpliconSeq_Decontamination/DecontPipeline/refs/";
```

```
### Step 0. Prepare working folders
my $command = "mkdir tmp/ data.out/";
print("$command\n");
system($command);

my $bsn = basename($file);
$bsn =~ s/\..*$//;

### Step 0. Rename contigs
my $command = "perl bin/NumberHeadsFasta.pl $file tmp/$bsn.fas
tmp/$bsn.idx";
print("$command\n");
system($command);

unless( -e "tmp/$bsn.blast"){
  ## Step 1. Run blast search
  my $command = "blastn -query tmp/$bsn.fas -db $db -task blastn -dust yes
-num_threads $nthreads -evalue $eval -perc_identity $pctsim -out
tmp/$bsn.blast -outfmt 6 -max_target_seqs 1";
  print("$command\n");
  system($command);

  if($mode =~ "CompleteSeq"){
    my $command = "perl bin/DeleteBlastDoublons2.pl tmp/$bsn.blast
tmp/$bsn.blast"; #clean blast outputs
    print("$command\n");
    system($command);
    }

  ### Step 2. Collect annotations
  my $command = "awk -F \'\\t\' \'{print \$2}\' tmp/$bsn.blast | awk -F
\'|\' \'{print \$4}\' > tmp/$bsn.gb";
  print("$command\n");
  system($command);
```

```
  open(IN, "tmp/$bsn.gb");

  my @accs;

  while(<IN>){

    chomp();

    push(@accs, $_);

    }

  my $entries = join(",", @accs);

  close(IN);


  my $command = "blastdbcmd -db $db -entry $entries -outfmt \"%a\t%t\" > 
tmp/$bsn.annot";

  print("$command\n");

  system($command);


  my $command = "./bin/blast2lca -names $refpath/names.dmp -nodes 
$refpath/nodes.dmp -dict $refpath/gi_taxid.bin -
levels=superkingdom:kingdom:phylum:order:family -order=true tmp/$bsn.blast 
--savemem > tmp/$bsn.taxo";

  print("$command\n");

  system($command);

  }


## Step 3. Collect contigs matching for a keyword of interest, extract hits 
or keep complete sequences

my $command = "perl bin/FetchFasta\_KeyWord.pl tmp/$bsn.fas tmp/$bsn.taxo 
tmp/$bsn.blast $keyword $mode data.out/$bsn.$keyword.$mode.clean";

print("$command\n");

system($command);


### Step 4. Put in perspective of annotations

my $command = "perl bin/CrossParseAnnots.pl tmp/$bsn.blast tmp/$bsn.taxo 
tmp/$bsn.annot tmp/$bsn.idx data.out/$bsn.$keyword.$mode.clean 
data.out/$bsn.$keyword.$mode.infos";

print("$command\n");

system($command)
```

Appendix S2 Removing contaminant sequences from reference catalog.

The following bash script takes as input a set of contigs and a file containing contaminant sequences. The input is blasted against the contaminant file and matching sequences are removed.

```
### Get script arguments
my $inputfile = $ARGV[0];
my $contaminantfile = $ARGV[1];
my $prefix = $ARGV[2];

### Important parameters
my $minlength = 80; #minimum length of contaminants sequences in pb
my $eval = 0.1; #min e-value for blast search
my $format = 8; #format for blastall output file

### Settings
my $bsn = $prefix;

### Prepare working folders
my $command = "mkdir tmp/ data.out/";
print("$command\n");
system($command);

### Rename input contigs file
my $command ="bash bin/bbmap/rename.sh in=$inputfile
out=tmp/$bsn.Rename.fasta prefix=$bsn";
print("$command\n");
system($command);

### Keep only contaminants sequences longer then n pb
my $command ="perl bin/Removesmall.pl $minlength $contaminantfile >
tmp/$bsn.Contaminants.Filtered.fasta";
print("$command\n");
system($command);

### Formating contaminant catalog into blast database format
```

```
my $command ="formatdb -t $bsn.db -i tmp/$bsn.Contaminants.Filtered.fasta -
p F -o F -a F -b F -e F -s F -V F";

print("$command\n");

system($command);


### Blast search with contaminant file as database

my $command ="blastall -p blastn -d tmp/$bsn.Contaminants.Filtered.fasta -i
tmp/$bsn.Rename.fasta -e $eval -m $format -o
tmp/$bsn.Matching.Sequences.csv -F F -I F -g T -J F -T F -U F -n F -V F -s
F";

print("$command\n");

system($command);


### Create a txt file with only the name of the matching sequences

my $command ="cut -f 1 tmp/$bsn.Matching.Sequences.csv >
tmp/$bsn.Matching.Names.txt";

print("$command\n");

system($command);


### Remove duplicate from matching sequences ID

my $command ="sort tmp/$bsn.Matching.Names.txt | uniq >
tmp/$bsn.Matching.Names.Unique.txt";

print("$command\n");

system($command);


### Remove matching contaminant sequences in the original contig file

my $command ="bash bin/bbmap/filterbyname.sh in=tmp/$bsn.Rename.fasta
out=data.out/$bsn.Filtered.fasta names=tmp/$bsn.Matching.Names.Unique.txt";

print("$command\n");

system($command)
```

Appendix S3 Simulation of population decline in Finges population.

The R-script takes as an input the minor allele frequencies of Finges 1940 and Finges 1950 populations. The parameters to set are the initial and final population sizes, the number of generations and the rate of decline.

```
###Initial minor allele frequency table
##Format : first column with SNPs IDs, second column with the minor allele
frequency value.
pobs = input final #Values after decline
p0 = input initial #Values before decline

###Preparing dataset
p0_sim = sample(p0, 1000, T, p0) #Sampling of 1000 SNPs
nloc = length(p0_sim) #Number of locus
loc = 1:nloc #Locus name
names(p0_sim) = loc #Adding the locus name

###Population parameters
N0 = 1000 #Initial population size
N_out = 1000 #Final population size
t = 10 #Number of generations
s = 0.8 #Survival rate
n = 900 #Number of individual removed from the population at each
generation

###########
## Model ##
###########

N_out = numeric(length(t))
N_out[1] = N0
p_out = p0_sim
p = matrix(nrow = nloc, ncol = t)
for(i in 1:t){
  print(i)
##At this step, you can choose between different type of decay
#Exponential weak
  N_out[i + 1] = round(N_out[i]*s)
```

```
#Linear
 #N_out[i + 1] = round(N_out[i]-n)
#Exponential strong
 #N_out[i + 1] = round(N_out[i]*(s^i))

  for(l in 1:nloc){
    a = sample(0:1, 2*N_out[i + 1], T, c(1-p_out[l],p_out[l]))
    p_out[l] = sum(a==1)/(2*N_out[i + 1])
    p[l,i] = p_out[l] #Matrix with minor allele frequency
  }
}

##Cleaning new SNPs matrix
p_out_final = subset(p_out, p_out != "0" & p_out !="1" & p_out < 0.5)
p_out_final = sample(p_out_final, length(p0), F)
```
